# Anion stabilised *hypercloso*-hexaalane Al_6_H_6_

**DOI:** 10.1038/s41467-018-05504-x

**Published:** 2018-08-06

**Authors:** Simon J. Bonyhady, David Collis, Nicole Holzmann, Alison J. Edwards, Ross O. Piltz, Gernot Frenking, Andreas Stasch, Cameron Jones

**Affiliations:** 1 School of Chemistry, Monash University, PO Box 23, Clayton, VIC 3800 Australia; 2 Scientific Computing Department, STFC Rutherford Appleton Laboratory, Harwell Oxford, Didcot, OX11 0QX UK; 3 Australian Centre for Neutron Scattering, Australian Nuclear Science and Technology Organisation, Locked Bag 2001, Kirrawee DC, NSW 2232 Australia; 4 Fachbereich Chemie, Philipps-Universität Marburg, 35032 Marburg, Germany; 5 Present Address: EaStCHEM School of Chemistry, University of St Andrews, North Haugh, KY16 9ST St Andrews UK

## Abstract

Boron hydride clusters are an extremely diverse compound class, which are of enormous importance to many areas of chemistry. Despite this, stable aluminium hydride analogues of these species have remained staunchly elusive to synthetic chemists. Here, we report that reductions of an amidinato-aluminium(III) hydride complex with magnesium(I) dimers lead to unprecedented examples of stable aluminium(I) hydride complexes, [(^Ar^Nacnac)Mg]_2_[Al_6_H_6_(Fiso)_2_] (^Ar^Nacnac = [HC(MeCNAr)_2_]^−^, Ar = C_6_H_2_Me_3_-2,4,6 Mes; C_6_H_3_Et_2_-2,6 Dep or C_6_H_3_Me_2_-2,6 Xyl; Fiso = [HC(NDip)_2_]^−^, Dip = C_6_H_3_Pr^*i*^_2_-2,6), which crystallographic and computational studies show to possess near neutral, octahedral *hypercloso*-hexaalane, Al_6_H_6_, cluster cores. The electronically delocalised skeletal bonding in these species is compared to that in the classical borane, [B_6_H_6_]^2−^. Thus, the chemistry of classical polyhedral boranes is extended to stable aluminium hydride clusters for the first time.

## Abstract (web summary)

While polyhedral boron hydride complexes have found application in a number of diverse fields, the isolation of stable aluminium analogues remains highly challenging. Here, Jones and colleagues demonstrate that reduction of an amidinato-aluminum(III) hydride complex with magnesium(I) dimers affords stable aluminium(I) hydride compounds.

## Introduction

The binary hydrides of boron, i.e. boranes (typically [B_*x*_H_*y*_]^*z*−^, *x* ≤ *y*, *z* = 0–2), are of enormous importance to chemistry from both fundamental and applications standpoints. The vast majority of these species are low oxidation state boron cluster compounds, which exhibit an enormous array of structural types^1^. The understanding of the structures of such clusters required the early development of revolutionary theories on chemical bonding (e.g. Wade–Mingos rules for electron counting)^2,3^, which ultimately led to boranes finding applications in areas as diverse as synthesis^4^, rocket fuel technology^5^ and medical science^6^.

It is remarkable that aluminium, boron’s neighbour in group 13, does not form any isolable hydride cluster compounds, or indeed many binary hydride compounds at all, e.g. AlH_3_, H_2_Al(μ-H)_2_AlH_2_ and [AlH_4_]^−^^7^. With that said, a handful of transient, low oxidation state alane cluster compounds have been studied in the gas phase, and some, e.g. Al_4_H_6_, have been shown to have fleeting stability^8–11^. Given that numerous ligand substituted, metalloid aluminium cluster compounds, e.g. [Al_77_{N(SiMe_3_)}_20_]^2−^, have been reported to be stable at, or close to, room temperature^12,13^, it seemed that related low-valent aluminium hydride clusters might be ultimately accessible under the right preparative conditions. As a prelude to realising this goal, we have synthesised the first stable binary low oxidation state aluminium hydride fragments, viz. [Al_2_H_6_]^2−^ and Lewis base stabilised Al_2_H_4_, by reduction of aluminium(III) hydride precursors with magnesium(I) dimers^14,15^.

Here, we report that related reductions of an amidinato-aluminium(III) hydride complex lead to unprecedented examples of stable aluminium(I) hydride complexes, [(^Ar^Nacnac)Mg]_2_[Al_6_H_6_(Fiso)_2_] (^Ar^Nacnac = [HC(MeCNAr)_2_]^−^, Ar = C_6_H_2_Me_3_-2,4,6 Mes; C_6_H_3_Et_2_-2,6 Dep or C_6_H_3_Me_2_-2,6 Xyl; Fiso = [HC(NDip)_2_]^−^, Dip = C_6_H_3_Pr^*i*^_2_-2,6), which possess near neutral *hypercloso*-hexaalane, Al_6_H_6_, cluster cores. Thus, this work represents a unique extension of the chemistry of classical polyhedral boranes to that of their alane analogues.

## Results

### Synthetic and spectroscopic studies

**Fig. 1:**
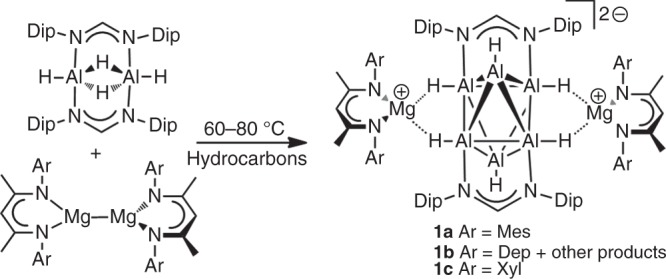
Formation of compounds **1** (Mes = C_6_H_2_Me_3_-2,4,6; Dep = C_6_H_3_Et_2_-2,6; Xyl = C_6_H_3_Me_2_-2,6). The compounds **1** are prepared by reduction of [{(μ-*N,N*-Fiso)Al(H)(μ-H)}_2_] with magnesium(I) dimers

Treatment of benzene, toluene, cyclohexane or hexane solutions of the formamidinato-aluminium(III) hydride complex, [{(μ-*N,N*-Fiso)Al(H)(μ-H)}_2_]^16^, with 1.2–2.0 equivalents of β-diketiminato ligated magnesium(I) dimers, [{(^Ar^Nacnac)Mg}_2_] (Ar = Mes, Dep or Xyl)^17–19^ (see Supplementary Materials and Supplementary Figs. 1–5), at elevated temperatures (typically 60–80 °C) reproducibly afforded low yields (ca. 5–20%) of the deep red crystalline aluminium(I) hydride cluster compounds **1** (Fig. 1), upon cooling the reaction solutions to ambient temperature. On several occasions, a number of low yielding colourless crystalline by-products were isolated from the reaction mixtures, including [(Fiso)Mg(^Dep^Nacnac)], [(Fiso)_2_AlH]^16^, [{(^Mes^Nacnac)Mg(μ-H)}_2_]^15^ and the dialanate salt, [{(^Mes^Nacnac)Mg}_2_(μ-H)]_2_[H_3_Al–AlH_3_]^14^ (see Supplementary Methods and Supplementary Figs. 6–9). The nature of these by-products suggests that the reductive mechanism for the formation of **1** could involve several intermediates and/or could compete with side reactions. In order to assess these possibilities, reactions that gave **1** at 70 °C were followed by ^1^H NMR spectroscopy. This revealed complex mixtures of products after several minutes heating, of which [(Fiso)Mg(Nacnac)] was identified in significant quantities (Supplementary Fig. 10). Definitive identification of products, other than those which were later isolated as crystalline solids, was not possible, and the mechanism of formation of **1** is not certain at this time.

To the best of our knowledge compounds **1** represent the first examples of isolated aluminium(I) hydride complexes, though mononuclear examples have recently been tentatively proposed as unstable intermediates in solution-based reactions^20^. The cluster compounds have negligible solubility in common deuterated solvents once crystallised, so no meaningful solution state spectroscopic data could be acquired for them. The most relevant solid state spectroscopic data (Supplementary Figs. 11, 12) for the compounds come from their infrared spectra, which exhibit single bands in the characteristic region for terminal Al–H stretching modes^7^ (e.g. **1a**: *ν* = 1798 cm^−1^). In addition, stronger bands are seen at lower wavenumber (e.g. **1a**: *ν* = 1648 cm^−1^) that possibly arise from a weakly bridging Al–H···Mg stretching mode, though these bands overlap with ligand stretching absorptions (see below). Noteworthy is the fact that the band at *ν* = 1798 cm^−1^ observed for **1a** is completely absent in the infrared spectrum of its hexa-deuteride analogue, **1a-D**, which was prepared by magnesium(I) reduction of [{(μ-*N,N*-Fiso)Al(D)(μ-D)}_2_]. The Al-D stretching band for **1a-D** should occur at ca. 1270 cm^−1^, but this is likely masked by strong ligand stretching modes in that region (Supplementary Fig. 12).

### Crystallographic studies

**Fig. 2:**
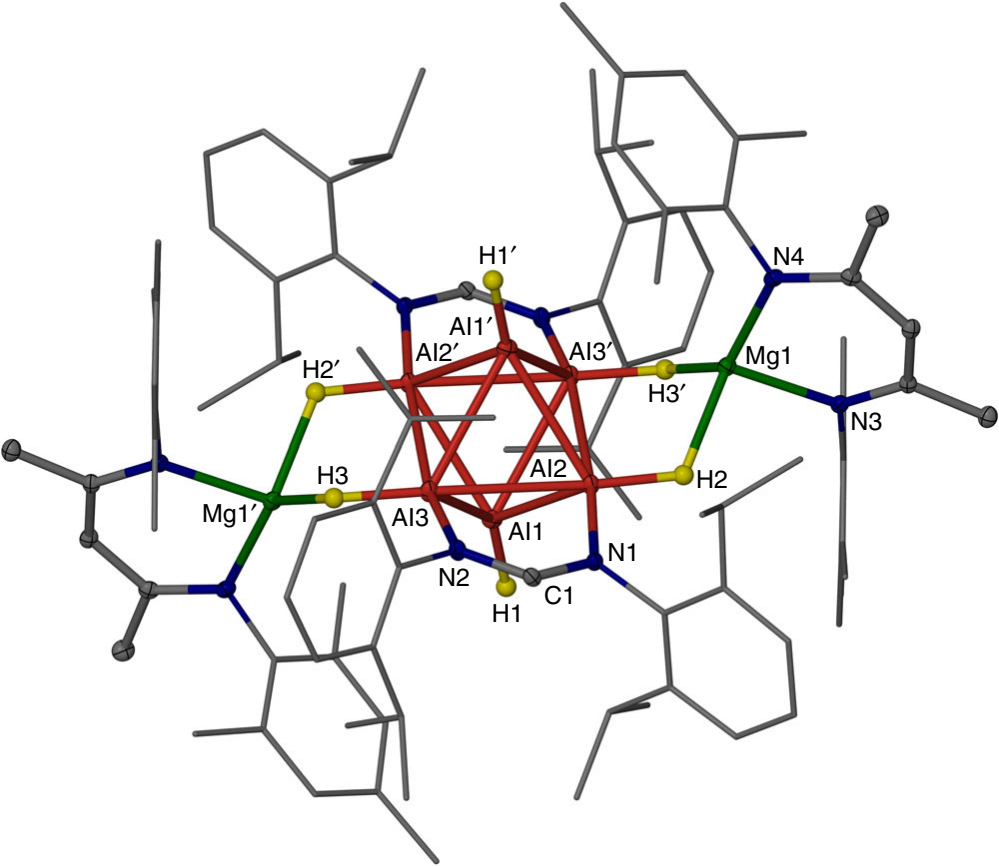
Molecular structure of **1a**. Hydrogen atoms, except hydrides, omitted. Ellipsoids shown at the 20% probability level, except aryl substituents, which are shown as wire frame. Selected bond lengths (Å): Al(1)–Al(2) 2.6317(15), Al(1)–Al(3) 2.6340(17), Al(1)–Al(2)′ 2.6472(15), Al(1)–Al(3)′ 2.6907(18), Al(2)–Al(3) 2.701(2), Al(2)–Al(3)′ 2.8257(14), Al(3)–N(2) 1.950(3), Al(2)–N(1) 1.925(3), Al(1)–H(1) 1.52(3), Al(2)–H(2) 1.55(3), Al(3)–H(3) 1.57(3), Mg(1)–H(2) 1.96(3), Mg(1)–H(3)′ 1.98(3). Symmetry operation: −*x* + 2, −*y* + 1, −*z*

All complexes **1** were crystallographically characterised and found to be isostructural, so only the molecular structure of **1a** is depicted in Fig. 2 (see Supplementary Methods, Supplementary Table 1 and Supplementary Figures 13 and 14). The hydride ligands of each cluster were located from difference maps and freely refined. A neutron diffraction study was also carried out on compound **1a**, which unambiguously confirmed the presence and connectivity of the six hydride ligands, and the absence of any other terminal, bridging or interstitial hydrides within the cluster core (see Supplementary Methods and Supplementary Figs. 15, 16). The compounds can be considered as having near neutral, distorted octahedral Al_6_H_6_ cores, opposing equatorial sides of which are coordinated by bridging, electronically delocalised formamidinate ligands. The remaining equatorial sides of the octahedron are bridged by [(^Mes^Nacnac)Mg]^+^ cations, which have weak interactions with the two hydride ligands that project from each side. Terminal hydride ligands coordinate to the apical aluminium centres of the cluster core, though these are slightly offset from the vector passing through the two aluminium centres to which they are coordinated, presumably for steric reasons. All of the Al–Al distances within the Al_6_ core lie in the known range for such bonds (mean: 2.72(12) Å, search of the Cambridge Crystallographic Database, February 2018), though the equatorial Al–Al distances (2.701(2) Å and 2.826(2) Å) are significantly longer than those between all axial and equatorial aluminium centres (2.631(2)–2.691(2) Å). The shorter of the equatorial Al–Al interactions are, not surprisingly, those which are bridged by the formamidinate ligands.

### Electronic structure and computational studies

The neutral distorted, octahedral Al_6_H_6_ cluster cores of **1** somewhat resemble the structure of the classical polyhedral borane, *closo*-[B_6_H_6_]^2−^ ^1^, despite their aforementioned elongated equatorial Al–Al interactions. This is intriguing as Al_6_H_6_ can be viewed as having 12 (i.e. 2*n*) valence electrons (i.e. 2 from each Al vertex) contributing to the skeletal Al–Al bonding of the cluster core. As such, it would be expected to have a more unsymmetrical, capped structure than the 14 skeletal valence electron (2*n* + 2) *closo*-[B_6_H_6_]^2−^, according to Wade–Mingos rules^2,3^. Indeed, computational studies have predicted a number of more open and unsymmetrical structures for Al_6_H_6_^21,22^, which are close in energy. Of course, in **1** the distorted octahedral geometry of this fragment is likely enforced by coordination to the amidinate ligands, which computational studies suggest do not add to the skeletal electron count (see below). For sake of comparison, isoelectronic B_6_H_6_ and Ga_6_H_6_, which have not been isolated experimentally, have been predicted to have capped trigonal bipyramidal *hypercloso*-structures, with all hydrides terminal^23,24^. Also worthy of mention are several ligand substituted analogues of Al_6_H_6_, e.g. B_6_(NMe_2_)_6_^25^ and Ga_6_{SiMe(SiMe_3_)_2_}_6_^24^, which possess distorted octahedral structures with several elongated E–E bonds, not dissimilar to the situation in **1**. In the case of Ga_6_{SiMe(SiMe_3_)_2_}_6_, calculations on the model compound Ga_6_H_6_ suggest that this can be attributed to a Jahn–Teller distortion arising from loss of degeneracy of the *t*_*2g*_ HOMOs of *closo*-[Ga_6_H_6_]^2−^ upon removal of two electrons from that dianion^24^.

In order to shed light on the nature of the bonding in the Al_6_H_6_ core of **1**, DFT calculations (theory level: RI-BP86/def2-TZVPP) were carried out on a cut-down model of the cluster compounds, viz. [(^Me^Nacnac)Mg]_2_[Al_6_H_6_(^H^Fiso)_2_] **1′** (^Me^Nacnac = [HC(MeCNMe)_2_]^−^, ^H^Fiso = [HC(NH)_2_]^−^) (Supplementary Methods). The geometry of the complex (Supplementary Fig. 7 and Supplementary Table 2) optimised to be similar to those of **1**, including a distorted octahedral Al_6_H_6_ core with somewhat shorter Al_ax_–Al_eq_ bonds (2.650–2.668 Å) than Al_eq_–Al_eq_ distances (2.718–2.819 Å). Reassuringly, the calculated infrared spectrum of **1′** (Supplementary Table 3) exhibits terminal and bridging Al–H stretching bands (*ν* = 1797 cm^−1^ (m) and 1649 cm^−1^ (s) respectively) that are very close to the experimental values for **1a**, thus supporting the use of **1′** as a model for **1**. The charges on the whole Al_6_H_6_ fragment (−0.67), both ^H^Fiso ligands (−1.10) and both (^Me^Nacnac)Mg fragments (+1.76) (Supplementary Table 4), indicate that **1′** is best viewed as an anion coordinated, near neutral *hypercloso*-Al_6_H_6_ cluster, having weak hydride bridges to [(^Me^Nacnac)Mg]^+^ cationic units. The calculated Wiberg bond indices (WBI) for the Al_ax_–Al_eq_ bonds (0.63–0.66) (Supplementary Table 5) are suggestive of relatively strong bonding interactions, while the WBIs for the Al_eq_-Al_eq_ interactions are much smaller (0.23–0.31). In line with this result is the fact that no bond critical points were found between the equatorial aluminium centres (Supplementary Fig. 8). Calculations on the [Al_6_H_6_(^H^Fiso)_2_]^2−^ dianion, in the absence of the [(^Me^Nacnac)Mg]^+^ cations, showed this fragment to be stable, with a geometry similar to that in the full contact ion compound (Supplementary Fig. 17 and Supplementary Table 2). This, combined with the fact that the uncoordinated Al_6_H_6_ octahedral unit was calculated to be an unstable entity in the electronic singlet state, confirms that the *hypercloso*-Al_6_H_6_ moiety of **1′** is stabilised by coordination to the ^H^Fiso anions.

**Fig. 3:**
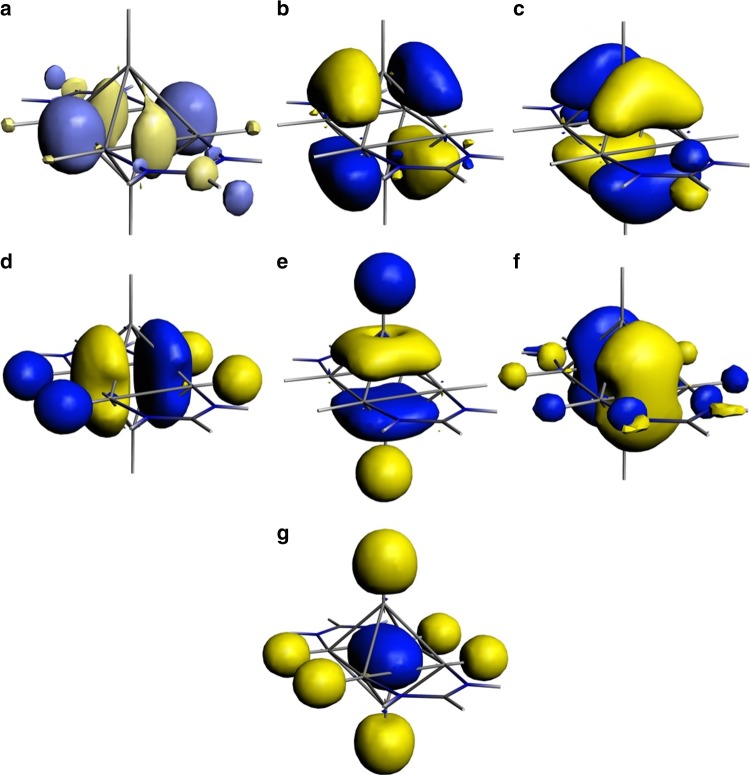
Representations of the aluminium-based MOs of [Al_6_H_6_(^H^Fiso)_2_]^2−^. **a** LUMO+7 (5.64 eV), **b** HOMO (3.42 eV), **c** HOMO-1 (3.30 eV), **d** HOMO-2 (1.96 eV), **e** HOMO-3 (1.84 eV), **f** HOMO-4 (1.70 eV), **g** HOMO-9 (−0.01 eV)

The electronic structure of the [Al_6_H_6_(^H^Fiso)_2_]^2−^ dianion was calculated and found to be similar to that of the full contact ion compound (Supplementary Figs. 19–21), so only the former is displayed in Fig. 3. There are seven Al-based molecular orbitals (MOs) on the dianion, six of which are filled, in line with the view that the cluster is a 12 skeletal valence electron species. None of these MOs are degenerate, but they do closely resemble the seven filled cluster based MOs for *closo*-[B_6_H_6_]^2−^ (triply degenerate *t*_2g_ and *t*_1u_ orbital sets, and *a*_1g_ orbital)^2^, and thus display significant electronic delocalisation over the Al_6_ core. The lack of degeneracy of the Al-based MOs of [Al_6_H_6_(^H^Fiso)_2_]^2−^ arises from the lower symmetry, and lower skeletal electron count, of the dianion relative to those of *closo*-[B_6_H_6_]^2−^. Interestingly, the LUMO+7 (lower energy empty MOs are ligand based) of [Al_6_H_6_(^H^Fiso)_2_]^2−^ resembles the degenerate *t*_2g_ HOMO of *closo*-[B_6_H_6_]^2−^ which exhibits the most analogous, *quasi*-equatorial B–B bonding character. This goes a long way to explaining the weak Al_eq_–Al_eq_ interactions in **1′**. The HOMO and HOMO-1 of [Al_6_H_6_(^H^Fiso)_2_]^2−^ are reminiscent of the other two *t*_2g_ orbitals of *closo*-[B_6_H_6_]^2−^, while the HOMO-2, HOMO-3 and HOMO-4 show similarities with the *t*_1u_ orbitals of the borane. At lower energy is the HOMO-9 which corresponds to the *a*_1g_ orbital of *closo*-[B_6_H_6_]^2−^. No Al-based MO exhibits significant contributions from the ^H^Fiso anions, the lone pairs of which are polarised towards their *N*-centres, and should therefore not be included in the counting of electrons contributing to Al–Al bonding within the cluster core. This view is supported by results of an energy decomposition analysis of the intrinsic interactions between the Al_6_H_6_ core and the amidinate ligands in [Al_6_H_6_(^H^Fiso)_2_]^2−^ (Supplementary Fig. 22 and Supplementary Table 6). Calculations of the NICS values of **1′** at the centre of the Al_6_H_6_ core suggest that the cluster exhibits significant 3-dimensional aromaticity (NICS_iso_ = −12.49 ppm, NICS_zz_ = −45.74 ppm) (Supplementary Table 7), as is common for polyhedral boranes^1^.

## Methods

### General

Experiments were carried out under a dry, oxygen-free dinitrogen atmosphere using Schlenk-line and glove-box techniques. All solvents and reagents were rigorously dried and deoxygenated before use. Compounds were variously characterised by elemental analyses, NMR, FTIR, and Raman spectroscopies, single crystal X-ray diffraction studies, and DFT calculations. Further details are available in Supplementary Methods.

### Preparation of [(^Xyl^Nacnac)MgI(OEt_2_)]

A freshly prepared solution of MeMgI (28.4 mmol) in diethyl ether (80 mL) was added over 20 min to a stirred solution of ^Xyl^NacnacH^26^ (8.08 g, 26.4 mmol) in diethyl ether (100 mL) at −20°C, yielding a colourless precipitate. The suspension was warmed to room temperature and stirred for 1 h after which time the precipitate of the title compound was collected by filtration. The supernatant solution was concentrated to ca. 40 mL and cooled to −30 °C to afford a second crop (12.86 g, 90%). M.P. 195–197 °C (decomp.); ^1^H NMR (400 MHz, 298 K, C_6_D_6_) *δ* = 0.44 (br, 6H; OCH_2_C*H*_3_), 1.54 (s, 6H; NCC*H*_3_), 2.07 (br, 6H; *ortho*-C*H*_3_), 2.64 (br, 6H; *ortho*-C*H*_3_), 3.11 (br, 4H; OC*H*_2_CH_3_), 4.86 (s, 1H; C*H*), 6.75–7.15 (m, 6H; Ar-*H*); ^13^C{^1^H} NMR (100 MHz, 298 K, C_6_D_6_) *δ* = 13.0 (NC*C*H_3_), 18.7 (*ortho*-*C*H_3_), 21.1 (*ortho*-*C*H_3_), 23.4 (OCH_2_*C*H_3_), 65.9 (O*C*H_2_CH_3_), 95.3 (*C*H), 124.7, 129.6, 131.5, 147.7 (Ar-*C*), 168.8 (N*C*CH_3_); MS (EI 70 eV), *m*/*z* (%): 457.1 (MH^+^-OEt_2_, 5), 306.4 (^Xyl^NacnacH^+^, 100); IR (Nujol) *ν* (cm^−1^): 1518s, 1262m, 1215w, 1197m,1185m, 1148m, 1092m, 1021m, 996m, 857m, 848w, 775s, 758m, 636m. Note: A satisfactory reproducible microanalysis of the compound could not be obtained due to co-crystallisation of the product with small amounts (ca. 3%) of the β-diketimine, ^Xyl^NacnacH, which could not be removed after several recrystallisations.

### Preparation of [{(^Xyl^Nacnac)Mg}_2_]

Toluene (80 mL) and diethyl ether (ca. 2 mL) were added to [(^Xyl^Nacnac)MgI(OEt_2_)] (1.58 g, 2.40 mmol). The resultant solution was rapidly stirred over a sodium mirror (0.70 g, 30.4 mmol) for 5 days to yield a yellow/green suspension. This was filtered, the yellow filtrate concentrated to ca. 20 mL and placed at −30 °C overnight to give yellow crystals of the title compound. A second crop was isolated after further concentration and cooling of the supernatant solution (0.28 g, 29%). M.P. 180–181 °C (decomp.); ^1^H NMR (400 MHz, 298 K, C_6_D_6_) *δ* = 1.48 (s, 12H; NCC*H*_3_), 1.90 (br. s, 24H; *ortho*-C*H*_3_), 4.76 (s, 2H; C*H*), 6.85–7.10 (m, 12H; Ar-*H*); ^13^C{^1^H} NMR (100 MHz, 298 K, C_6_D_6_) *δ* = 19.2 (NC*C*H_3_), 23.1 (*ortho*-*C*H_3_), 95.3 (*C*H), 124.1, 128.4, 131.8, 148.0 (Ar-*C*), 166.3 (N*C*CH_3_); MS (EI 70 eV), *m*/*z* (%): 659.5 (MH^+^, 10); IR (Nujol) *ν* (cm^−1^): 1555s, 1520w, 1278m, 1262m, 1182m, 1094m, 1023m, 809w, 762m. Note: A satisfactory reproducible microanalysis of the compound could not be obtained due to co-crystallisation of the product with small amounts (ca. 5%) of the iodide bridged magnesium(II) dimer, [{(^Xyl^Nacnac)Mg(μ-I)}_2_], which could not be removed after several recrystallisations. The constitution of this co-crystallised mixture was confirmed by a poor quality crystal structure determination of [{(^Xyl^Nacnac)Mg}_2_], details of which are not reported here due to the low quality of the diffraction data.

### Preparation of [(^Mes^Nacnac)Mg]_2_[Al_6_H_6_(Fiso)_2_] (**1a**)

[{(μ-*N,N*-Fiso)Al(H)(μ-H)}_2_]^16^ (164 mg, 0.21 mmol) was added to a suspension of [{(^Mes^Nacnac)Mg}_2_]^27^ (300 mg, 0.42 mmol) in benzene (5 mL) in a grease-free Schlenk flask (20 mm diameter). The mixture was heated to 65 °C for 5 min or until a bright red solution formed. Allowing the solution to stand at room temperature for 4 days resulted in the deposition of red crystals of **1a** (20 mg, 18% based on aluminium). M.P. > 150 °C (decomp.) ^13^C{^1^H} NMR (75.5 MHz, 298 K, solid state) *δ* = 17.5, 20.6, 25.4, 27.7, 95.1, 123.8, 129.4, 131.5, 142.1, 146.4, 163.4, 167.4; MS (EI 70 eV), *m*/*z* (%): 335.5 (^Mes^NacnacH_2_^+^, 100), 365.5 (FisoH_2_^+^, 38); MALDI-TOF MS *m*/*z*: 335.5 (^Mes^NacnacH_2_^+^), 357.4 ((^Mes^Nacnac)Mg^+^), 365.5 (FisoH_2_^+^), 389.4 ((Fiso)Al–H^+^); IR (Nujol) *ν* (cm^−1^): 1798m (Al–H str.), 1648s (br, incl. Al–H str.), 1542vs, 1197m, 1176m, 1146s, 1097s, 1021s, 854s, 803s, 755s; Raman (solid under N_2_, 514 nm excitation, cm^−1^): *ν* = 3064m, 1352s, 1318m, 522m, 386m. A similar yield of **1a** was obtained when the reaction was conducted in cyclohexane (ca. 12 mL). Elemental analysis calculated for C_96_H_134_Al_6_Mg_2_N_8_·C_6_H_12_: C 72.29%, H 8.68%, N 6.61%; found: C 72.13%, H 8.55%, N 6.54%. Notes: (i) A few crystals of the known magnesium(II) hydride dimer, [{(^Mes^Nacnac)Mg(μ-H)}_2_]^15^, the known aluminium(III) hydride, [(Fiso)_2_AlH]^16^, and the new colourless dialanate salt, [{(^Mes^Nacnac)Mg}_2_(μ-H)]_2_[H_3_Al–AlH_3_], co-crystallised with **1a** from the reaction mixture. All were identified by X-ray crystallography (Supplementary Fig. 9). Insufficient amounts of [{(^Mes^Nacnac)Mg}_2_(μ-H)]_2_[H_3_Al–AlH_3_] were obtained to allow spectroscopic characterisation, but it is noteworthy that the 2,6-diethylphenyl substituted analogue of the compound, [{(^Dep^Nacnac)Mg}_2_(μ-H)]_2_[H_3_Al–AlH_3_], has been previously reported and fully characterised^14^. (ii) Reproducible low yield syntheses of **1a** (typically 5–20%) were achieved under a number of reaction conditions. For example, toluene, hexane, cyclohexane or benzene could be used as the reaction solvent, the reaction temperature was varied from ca. 60–80 °C, the reaction stoichiometry was varied from 1.2:1 to 2:1 ([{(^Mes^Nacnac)Mg}_2_]:[{(μ-*N,N*-Fiso)Al(H)(μ-H)}_2_]), and the time the reaction mixture was kept at elevated temperature varied from 5 to 25 min. The time required for the reaction depended strongly on the diameter of the reaction flask. (iii) Compound **1a** (and **1b-c**) have negligible solubility in common deuterated solvents once crystallised, so no meaningful solution state spectroscopic data could be acquired for them. Attempts to dissolve **1a** in *d*_*8*_-THF led to decomposition of the compound. (iv) Attempts were made to obtain solution state spectroscopic data on **1a** from red reaction solutions before it crystallised from those solutions. NMR spectroscopic data on those solutions showed complex product mixtures (Supplementary Fig. 10), while ESI mass spectroscopic analyses of the reaction solutions showed no ion that could be assigned to **1a** or its fragmentation products.

### Preparation of [(^Dep^Nacnac)Mg]_2_[Al_6_H_6_(Fiso)_2_] (**1b**)

[{(μ-*N,N*-Fiso)Al(H)(μ-H)}_2_]^16^ (102 mg, 0.13 mmol) was added to a suspension of [{(^Dep^Nacnac)Mg}_2_]^19^ (200 mg, 0.26 mmol) in benzene (2 mL) in a grease-free Schlenk flask (20 mm diameter). The mixture was heated to 65 °C for 5 min or until a bright red solution formed. Allowing the solution to stand at room temperature for 4 days resulted in the deposition of deep red crystals of **1b** (10 mg, 5% based on aluminium). M.P. > 150 °C (decomp.); IR (Nujol) *ν* (cm^−1^): 1834m (Al–H str.), 1633s (br, incl. Al–H str.), 1538s, 1366vs, 1338s, 1320s, 1261s, 1232m, 1177s, 1108s, 1023s, 946m, 802s. No solution state spectroscopic data could be obtained for the compound due to its negligible solubility in common organic solvents. A reproducible microanalysis of the compound could not be obtained due it its low yield and the fact that it co-crystallised with the known dialanate salt, [{(^Dep^Nacnac)Mg}_2_(μ-H)]_2_[H_3_Al–AlH_3_]^14^, and the new, colourless magnesium(II) complex, [(Fiso)Mg(^Dep^Nacnac)]. The latter compound was subsequently intentionally synthesised, spectroscopically characterised, and its X-ray crystal structure obtained (Supplementary Figure 8).

### Preparation of [(^Xyl^Nacnac)Mg]_2_[Al_6_H_6_(Fiso)_2_] (**1c**)

[{(μ-*N,N*-Fiso)Al(H)(μ-H)}_2_]^16^ (118 mg, 0.15 mmol) was added to a suspension of [{(^Xyl^Nacnac)Mg}_2_] (200 mg, 0.30 mmol) in benzene (3 mL) in a grease-free Schlenk flask (20 mm diameter). The mixture was heated to 65 °C for 5 min or until a bright red solution formed. Allowing the solution to stand at room temperature for 4 days resulted in the deposition of deep red crystals of **1c** (10 mg, 4% based on aluminium). M.P. > 150 °C (decomp.); IR (Nujol) *ν* (cm^−1^): 1829m (Al–H), 1623m (br incl. Al–H str.), 1593m, 1543vs, 1336s, 1322s, 1265m, 1257m, 1233m, 1185s, 1096m, 1031m, 951m, 934m, 843m, 802m, 762s, 698m. No solution state spectroscopic data could be obtained for the compound due to its negligible solubility in common organic solvents. A reproducible microanalysis of the compound could not be obtained due it its low yield and the fact that it co-crystallised with colourless crystalline compounds from which it could not be completely separated.

### Preparation of [(^Mes^Nacnac)Mg]_2_[Al_6_D_6_(Fiso)_2_] (**1a-D**)

[{(μ-*N,N*-Fiso)Al(D)(μ-D)}_2_] (165 mg, 0.21 mmol), prepared as per the procedure for [{(μ-*N,N*-Fiso)Al(H)(μ-H)}_2_]^16^ (see Supplementary Methods), was added to a suspension of [{(^Mes^Nacnac)Mg}_2_]^27^ (300 mg, 0.42 mmol) in benzene (5 mL) in a grease-free Schlenk flask (20 mm diameter). The mixture was heated to 65 °C for 5 min or until a bright red solution formed. Allowing the solution to stand at room temperature for 4 days resulted in the deposition of red crystals of **1a** (21 mg, 19% based on aluminium). M.P. > 150 °C (decomp.); IR (Nujol) *ν* (cm^−1^): 1665m, 1546s, 1388vs, 1366vs, 1338s, 1259s, 1231m, 1195m, 1146s, 1098s, 1022s, 856m, 803m, 754s, 698s; Raman (solid under N_2_, 514 nm excitation, cm^−1^): *ν* = 1353s, 1320m, 523w, 445m, 380w.

### Preparation of [(Fiso)Mg(^Dep^Nacnac)]

This compound was a by-product in the preparation of **1b**. It was subsequently intentionally synthesised as follows. Toluene (10 mL) was added to a solid mixture of [{(^Dep^Nacnac)Mg(μ-^*n*^Bu)}_2_]^19^ (0.390 g, 0.440 mmol) and FisoH (0.331 g, 0.907 mmol) at room temperature. The mixture was then stirred for 90 min at 40 °C to afford a colourless solution. The resultant solution was concentrated under reduced pressure to 3 mL, *n*-hexane (4 mL) was added, and the solution was stored at −30 °C overnight to afford colourless crystals of [(Fiso)Mg(^Dep^Nacnac)] (0.31 g, 47%). M.P.: gradually softens above 210 °C and takes on a yellow colour above 290 °C; ^1^H NMR (300 MHz, C_6_D_6_, 303 K): *δ* = 1.06 (d, ^3^*J*_H,H_ = 6.9 Hz, 24H; CH(C*H*_3_)_2_), 1.10 (t, ^3^*J*_H,H_ = 7.6 Hz, 12H; CH_2_C*H*_3_), 1.53 (s, 6H; NCC*H*_3_), 2.55 (dq, ^2,3^*J*_H,H_ = 15.0 Hz, 7.5 Hz, 4H; C*H*_2_CH_3_), 2.67 (dq, ^2,3^*J*_H,H_ = 15.0 Hz, 7.5 Hz, 4H; C*H*_2_CH_3_), 2.99 (sept, ^3^*J*_H,H_ = 6.9 Hz, 4H; C*H*(CH_3_)_2_), 4.92 (s, 1H; C*H*), 6.96–7.10 (m, 12H; Ar-*H*), 7.94 (s, 1H; N_2_C*H*); ^13^C{^1^H} NMR (75.5 MHz, C_6_D_6_, 303 K): *δ* = 13.8 (CH_2_*C*H_3_), 23.8 (NC*C*H_3_), 24.6 (*C*H_2_CH_3_), 25.0 (CH(*C*H_3_)_2_), 28.7 (CH(*C*H_3_)_2_), 96.0 (*C*H), 123.6, 123.9, 125.3, 125.9, 137.2, 142.5, 143.8, 147.4 (Ar-*C*), 169.9 (N*C*CH_3_), 171.5 (N_2_*C*H); IR (Nujol): *ν* (cm^−1^) = 1665m, 1595w, 1537s, 1531s, 1505m, 1462s, 1443s, 1390s, 1377s, 1321m, 1274s, 1266s, 1207m, 1181m, 1108m, 1031m, 1018m, 962w, 934m, 854m, 806m, 799m, 755s, 722m; elemental analysis calculated for C_50_H_68_N_4_Mg: C 80.13%, H 9.14%, N 7.48%; found: C 80.03%, H 9.17, N 7.43%.

### Data availability

The X-ray crystallographic coordinates for structures reported in this study have been deposited at the Cambridge Crystallographic Data Centre (CCDC), under deposition numbers 1830325–1830331. These data can be obtained free of charge from The Cambridge Crystallographic Data Centre via www.ccdc.cam.ac.uk/data_request/cif.

## Electronic supplementary material

Supplementary Information
