# Supplementary material for: Anion stabilised hypercloso-hexaalane Al6H6
Source: Nat Commun. 2018 Aug 6;9:3079. doi: 10.1038/s41467-018-05504-x (PMC6079018; doi:10.1038/s41467-018-05504-x)
Supplement: Supplementary file 1 — Supplementary Information [file 41467_2018_5504_MOESM1_ESM.pdf]

Anion Stabilised *hypercloso*-Hexaalane Al_6H_6

Simon J. Bonyhady *et al.*

Supplementary Figures

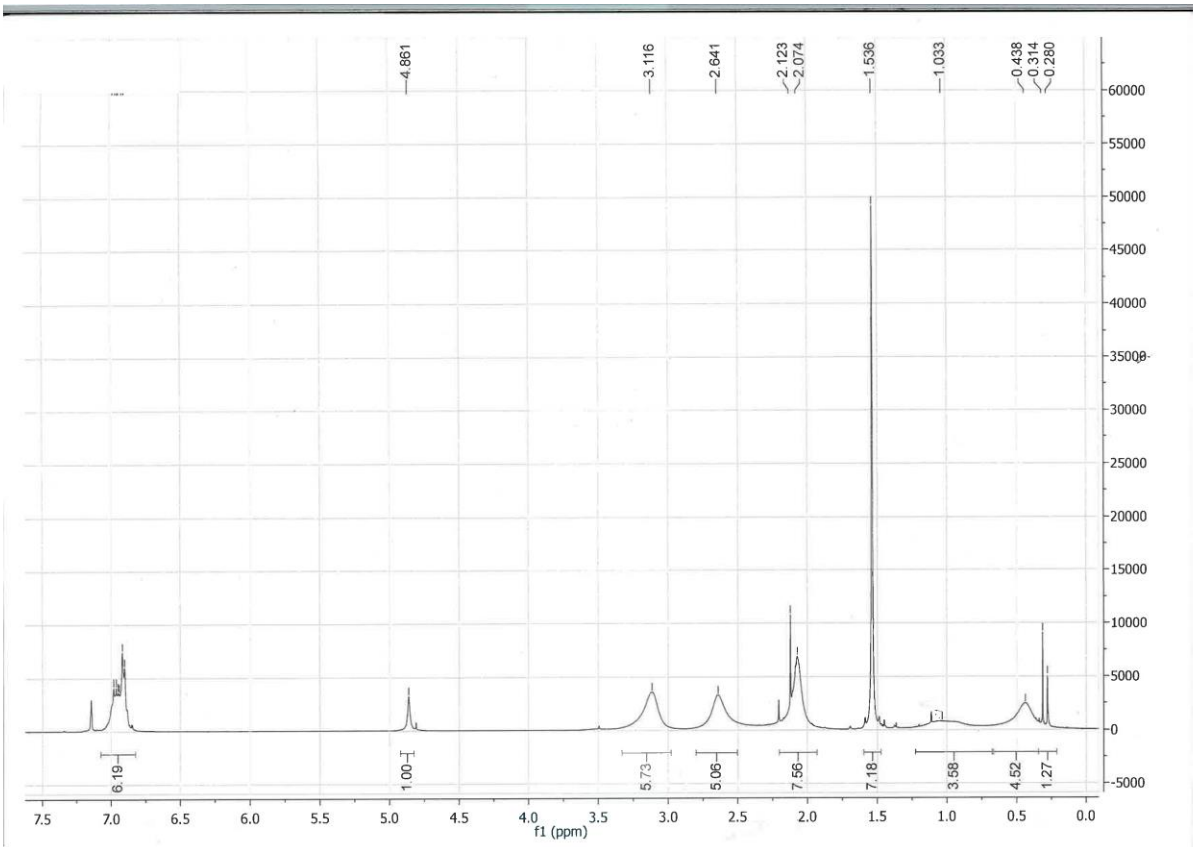

Supplementary Figure 1. ^{1}H NMR spectrum (400 MHz, C_6D_6) of [(^{Xyl}Nacnac)MgI(OEt_2)].

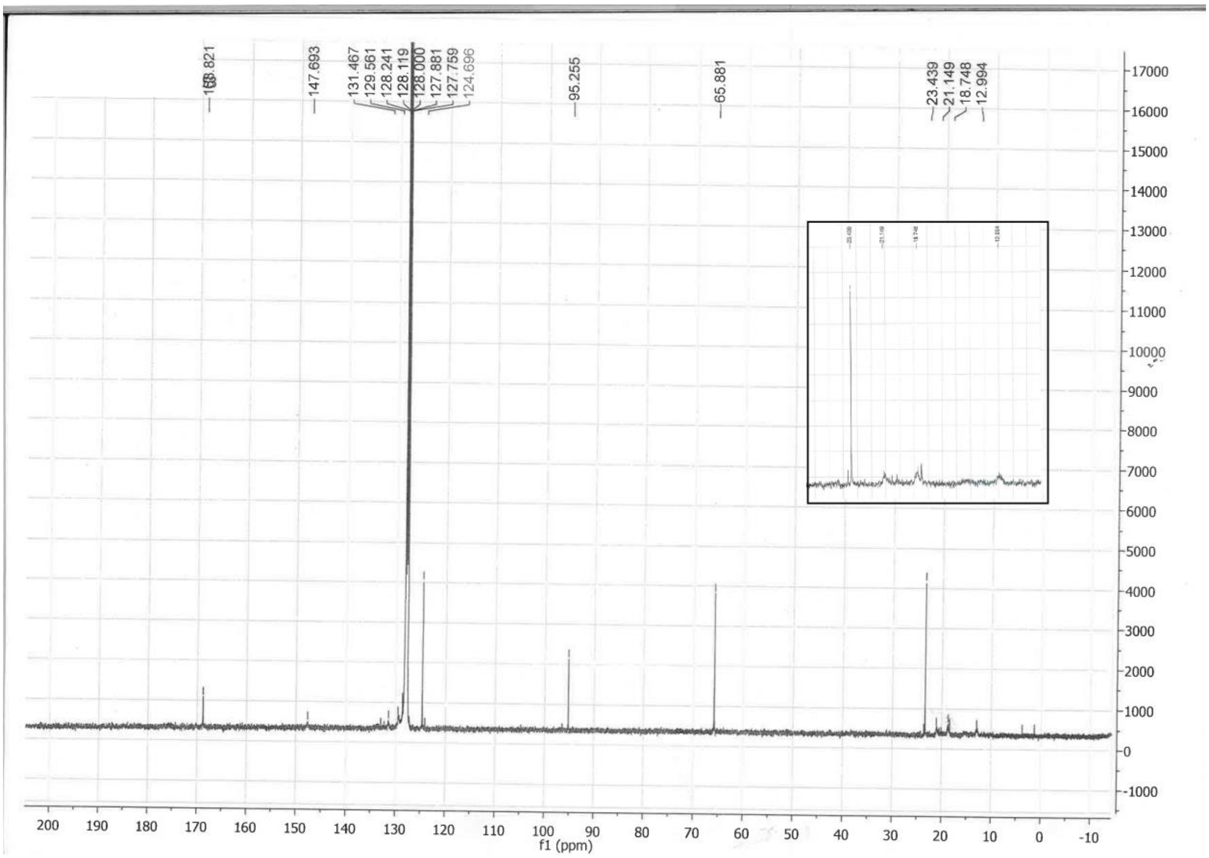

Supplementary Figure 2. $^{13}C\{^1H\}$ NMR spectrum (100 MHz, C_6D_6) of [(^{Xyl}Nacnac)MgI(OEt_2)], with an expansion of the 10-30 ppm chemical shift region inset.

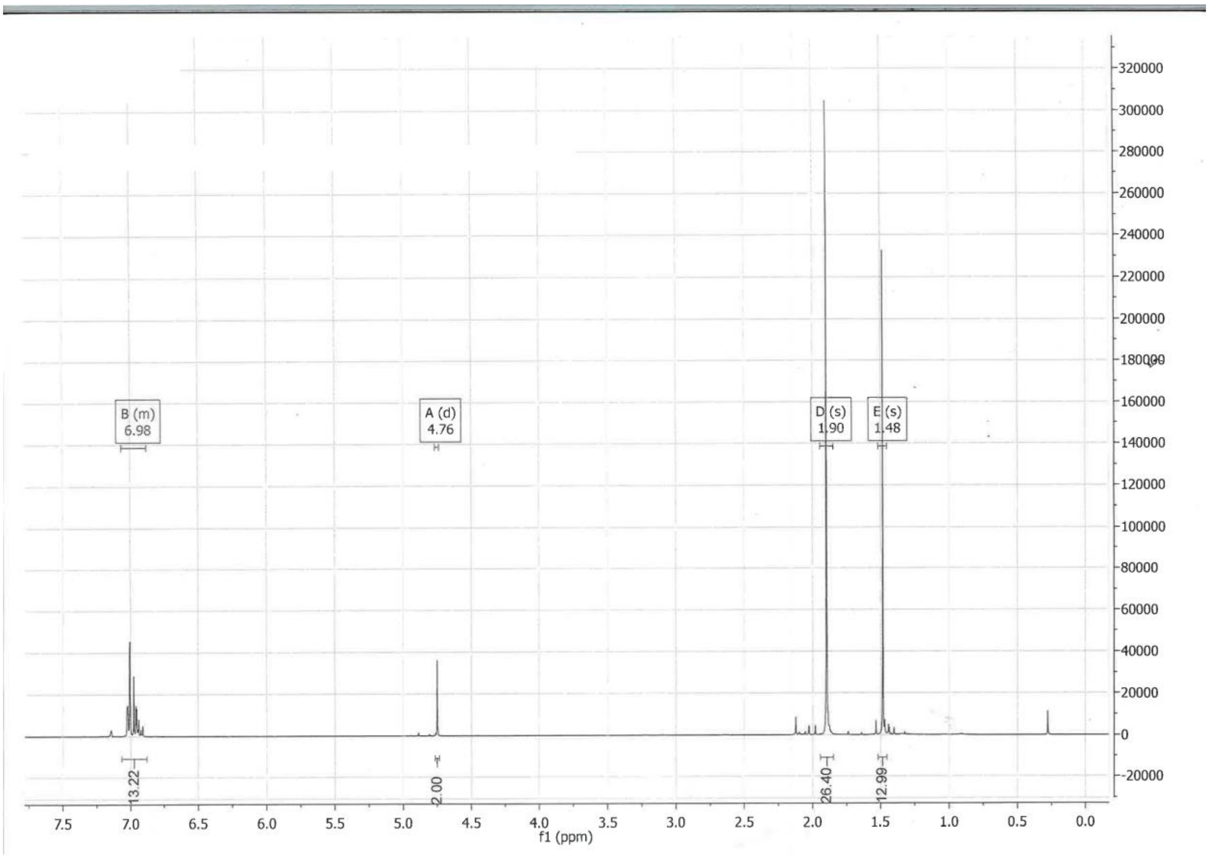

Supplementary Figure 3. ^{1}H NMR spectrum (400 MHz, C_6D_6) of [{(^{Xyl}Nacnac)Mg}$_2$].

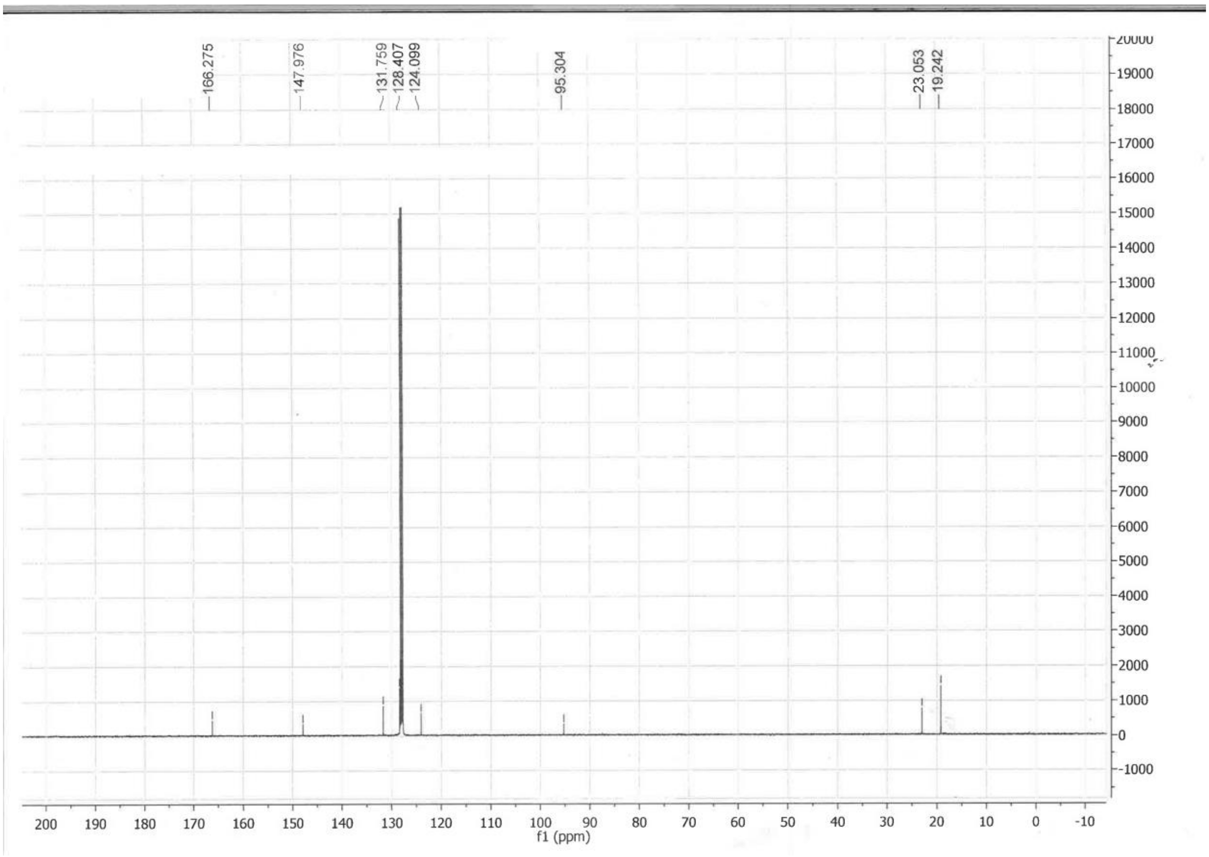

Supplementary Figure 4. $^{13}C\{^1H\}$ NMR spectrum (100 MHz, C_6D_6) of $[\{(^{Xyl}Nacnac)Mg\}_2]$.

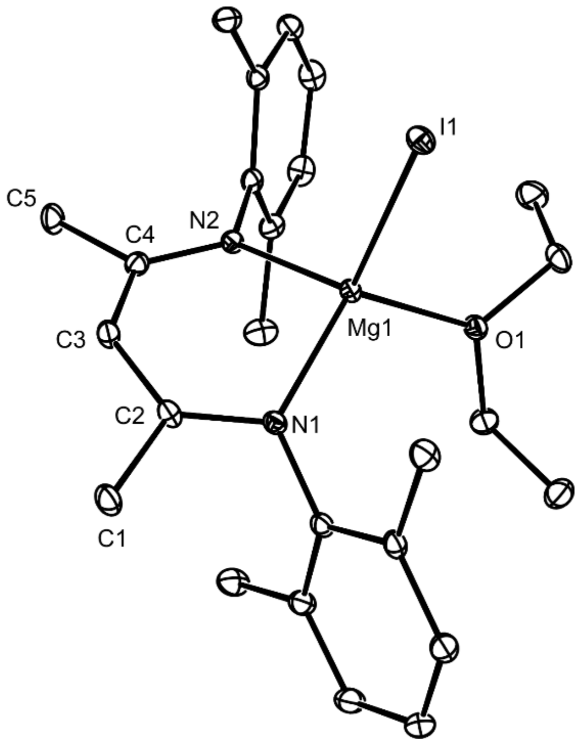

Supplementary Figure 5. Molecular structure of [(^{Xyl}Nacnac)MgI(OEt_2)] (hydrogen atoms omitted, ellipsoids shown at the 20% probability level). Selected bond lengths (Å) and angles (°): I(1)-Mg(1) 2.6867(8), Mg(1)-O(1) 2.0043(16), Mg(1)-N(1) 2.0263(18), Mg(1)-N(2) 2.0391(18), N(1)-Mg(1)-N(2) 94.18(7), O(1)-Mg(1)-I(1) 103.83(5).

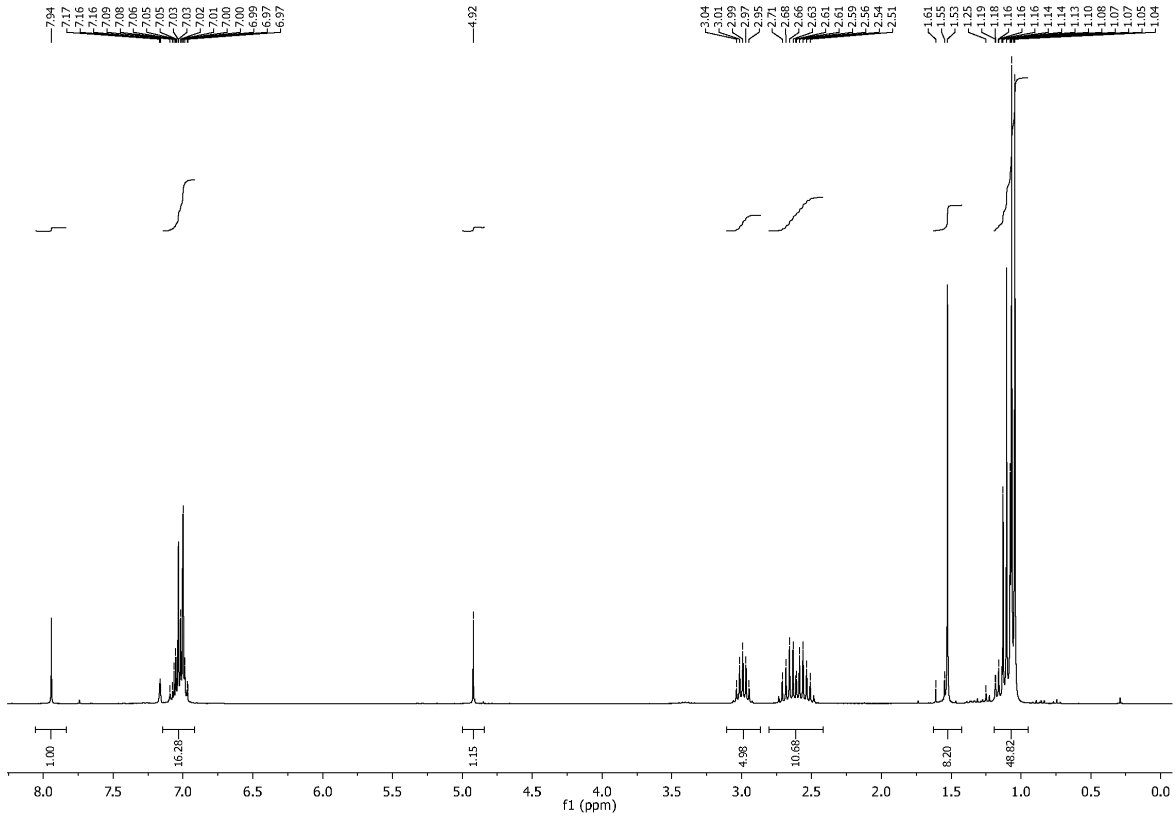

Supplementary Figure 6. ^{1}H NMR spectrum (300 MHz, C_6D_6) of [(Fiso)Mg(^{Dep}Nacnac)].

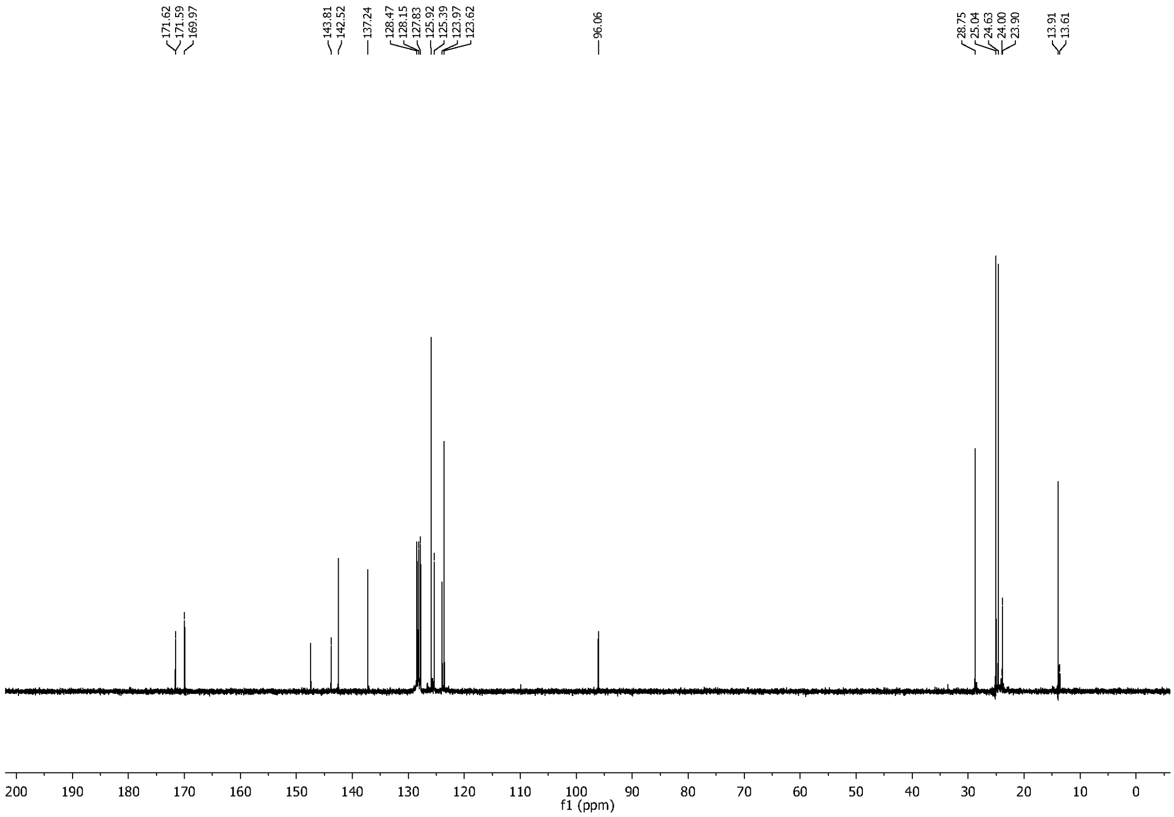

Supplementary Figure 7. $^{13}C\{^{1}H\}$ NMR spectrum (75.5 MHz, C_6D_6) of [(Fiso)Mg(^{Dep}Nacnac)].

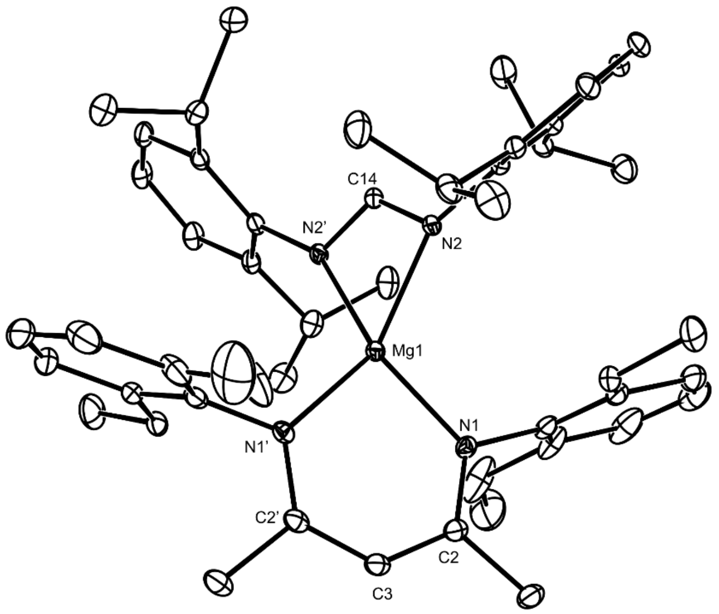

Supplementary Figure 8. Molecular structure of [(Fiso)Mg(^{Dep}Nacnac)] (hydrogen atoms omitted, ellipsoids shown at the 20% probability level). Selected bond lengths (Å) and angles (°): Mg(1)-N(1) 2.0336(19), Mg(1)-N(2) 2.1040(19), N(1)'-Mg(1)-N(1) 94.30(11), N(2)-Mg(1)-N(2)' 64.94(9), N(2)-C(14)-N(2)' 118.1(3). Symmetry operation: ' y, x, -z.

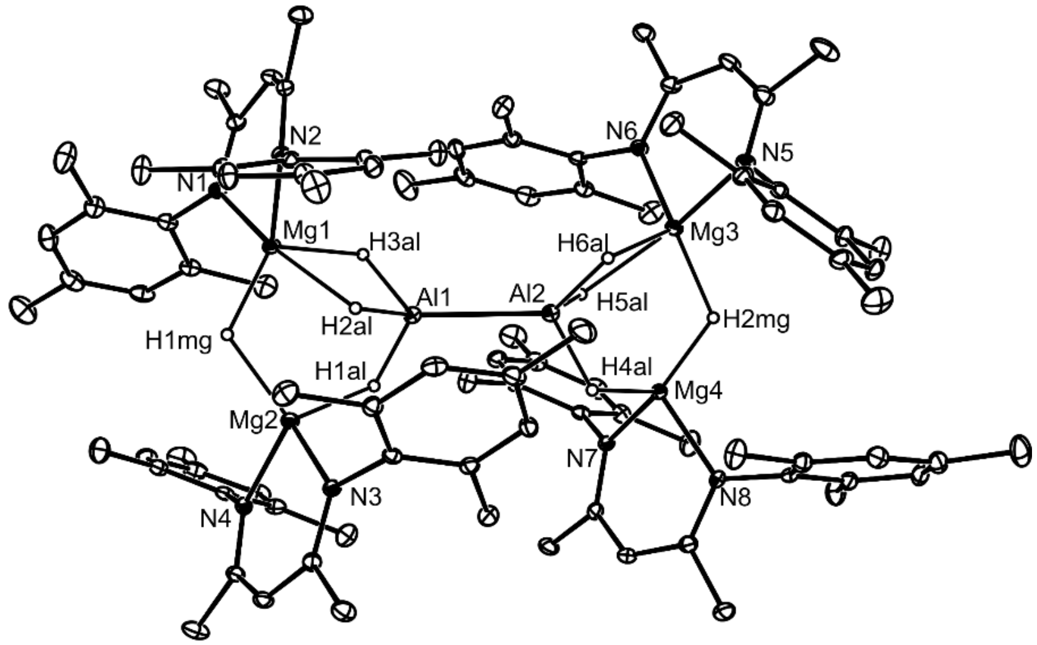

Supplementary Figure 9. Molecular structure of [{(^{Mes}Nacnac)Mg}$_2$(μ-H)]$_2$[H_3Al–AlH_3] (hydrogen atoms, except hydrides, omitted, ellipsoids shown at the 20% probability level). Selected bond lengths (Å): Al(1)-Al(2) 2.5145(13), Al(1)-H(1AL) 1.59(3), Al(1)-H(3AL) 1.65(3), Al(1)-H(2AL) 1.69(3), Mg(1)-H(3AL) 1.93(3), Mg(1)-H(2AL) 2.21(3), Mg(1)-H(1MG) 1.78(3), Al(2)-H(4AL) 1.59(3), Al(2)-H(5AL) 1.57(2), Al(2)-H(6AL) 1.63(2), Mg(2)-H(1AL) 1.94(3), Mg(2)-H(1MG) 1.90(3), Mg(3)-H(5AL) 2.20(2), Mg(3)-H(6AL) 2.07(3), Mg(3)-H(2MG) 1.91(3), Mg(4)-H(4AL) 1.95(2), Mg(4)-H(5AL) 2.33(2), Mg(4)-H(2MG) 1.71(3).

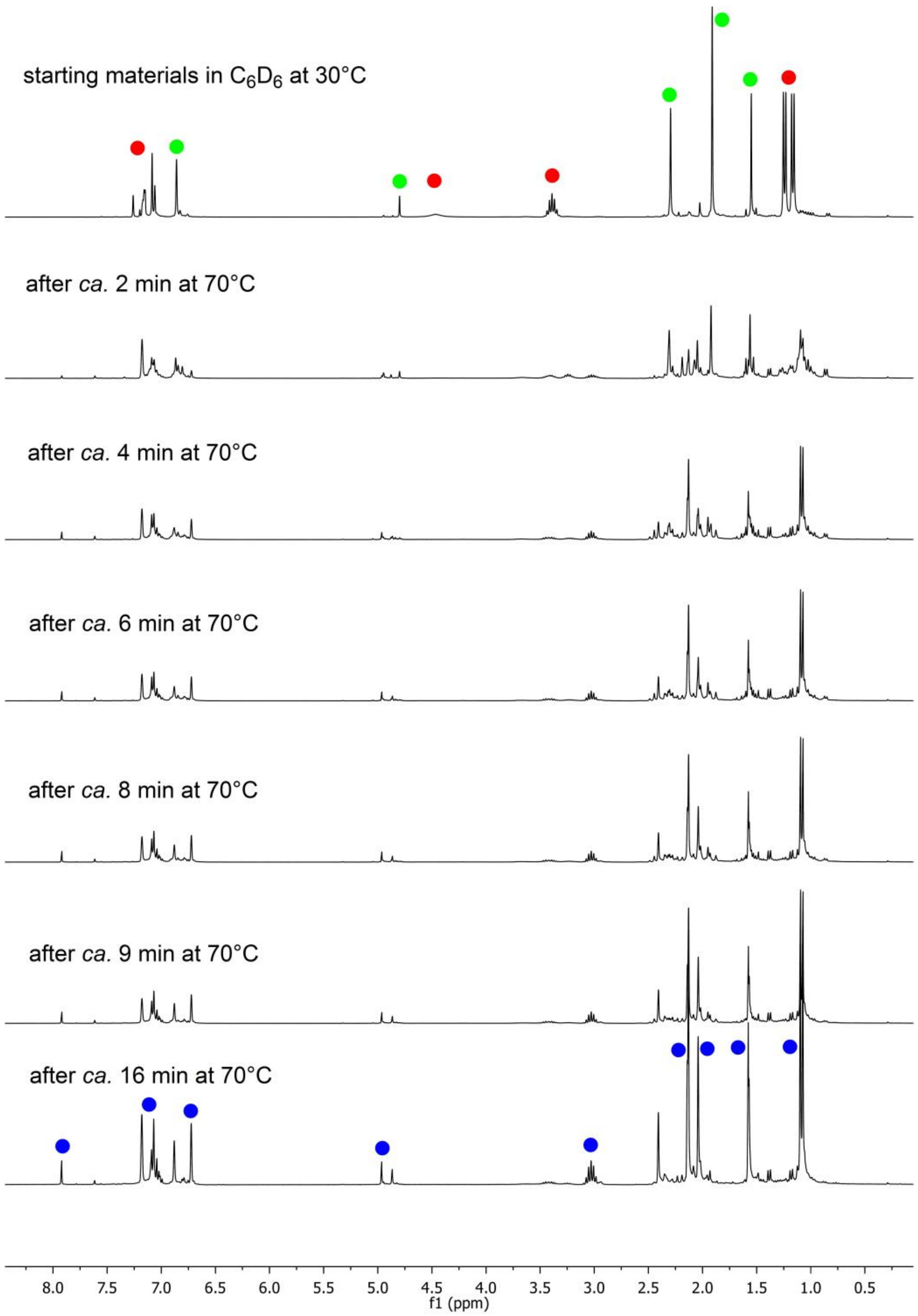

Supplementary Figure 10. *In-situ* reaction of [{(^{Mes}Nacnac)Mg}$_2$] (green dots) and [{(Fiso)AlH(μ-H)}$_2$] (red dots) (ratio *ca.* 1.8:1) at 70 °C in C_6D_6, followed by ^{1}H NMR spectroscopy (300.13 MHz). In the spectrum taken after 16 min, the peaks annotated with blue dots correspond to [(Fiso)Mg(^{Dep}Nacnac)]

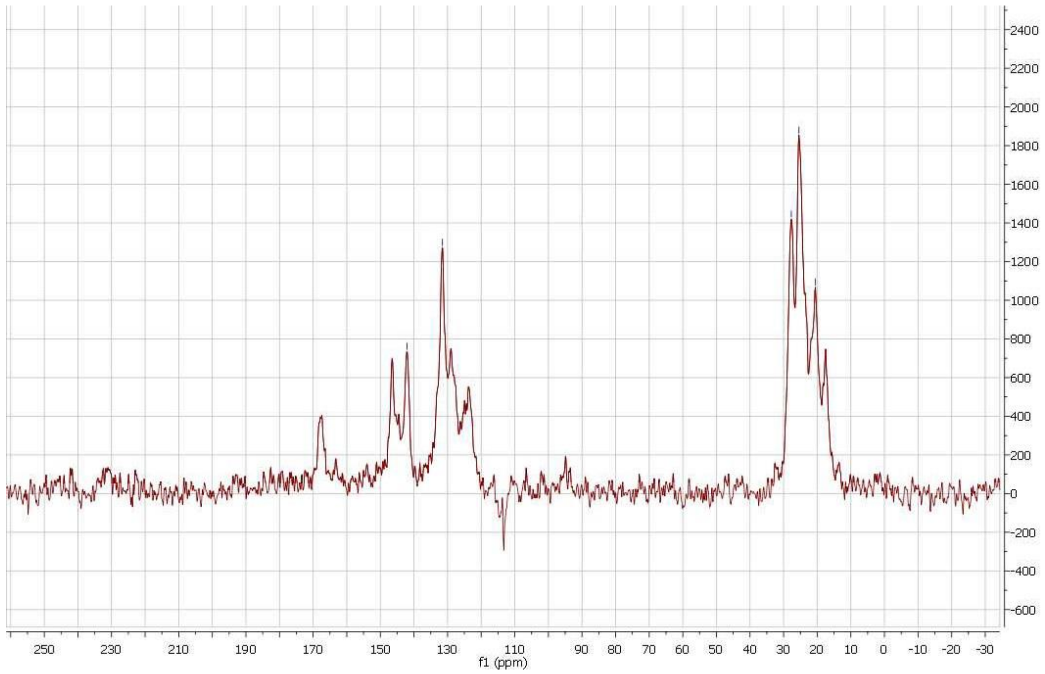

Supplementary Figure 11. Solid state $^{13}C\{^{1}H\}$ NMR spectrum (75.5 MHz) of $[(^{Mes}Nacnac)Mg]_2[Al_6H_6(Fiso)_2]$ (**1a**).

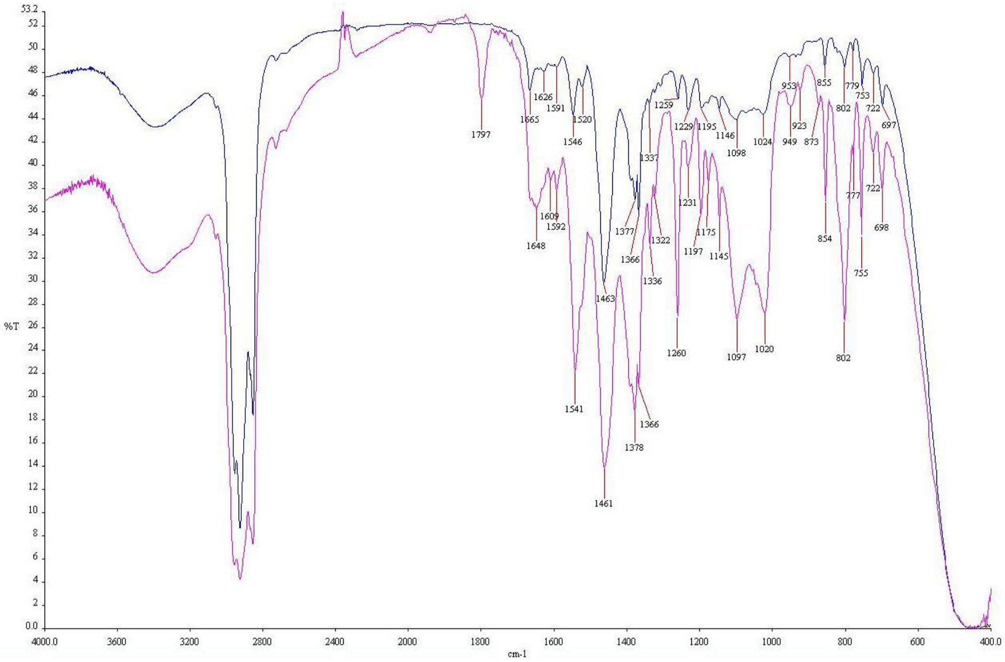

Supplementary Figure 12. Overlay of the infrared spectra (Nujol mulls) of **1a** (magenta), and **1a-D** (blue). Broad stretching band at $\tilde{\nu} \approx 3400$ cm^{-1} is due to trace moisture on NaCl plates. N.B.: The discrepancy in stretching band strength between the two samples is due to the preparation of a weaker sample of **1a-D.**

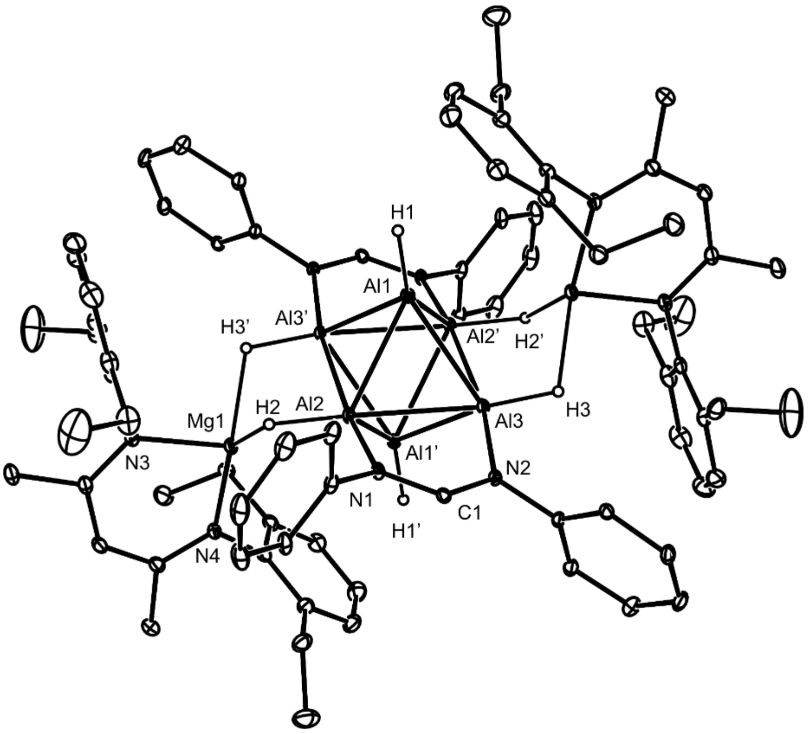

Supplementary Figure 13. Molecular structure of $[(^{Dep}\text{Nacnac})\text{Mg}]_2[\text{Al}_6\text{H}_6(\text{Fiso})_2]$ **1b** (hydrogen atoms, except hydrides, and isopropyl groups omitted, ellipsoids shown at the 20% probability level). Selected bond lengths (Å): Al(1)-Al(2) 2.6332(12), Al(1)-Al(3) 2.6436(12), Al(1)-Al(2)' 2.6533(12), Al(1)-Al(3)' 2.6924(11), Al(2)-Al(3) 2.6868(11), Al(2)-Al(3)' 2.8532(11), Al(1)-H(1) 1.531(18), Al(2)-H(2) 1.569(18), Al(3)-H(3) 1.548(18), Mg(1)-H(2) 2.03(3), Mg(1)-H(3)' 1.89(3). Symmetry operation: '-x+2, -y+1, -z.

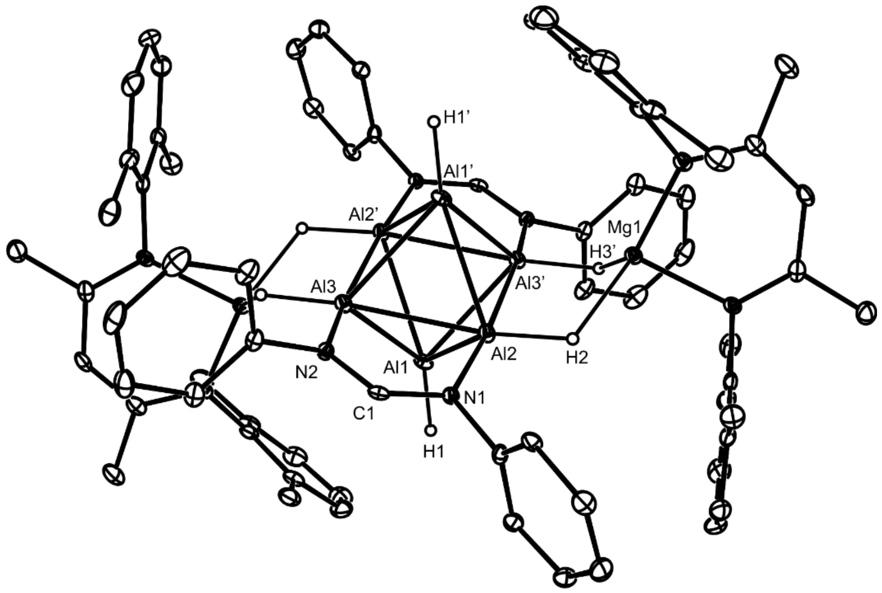

Supplementary Figure 14. Molecular structure of $[(^{Xyl}Nacnac)Mg]_2[Al_6H_6(Fiso)_2]$ **1c** (hydrogen atoms, except hydrides, and isopropyl groups omitted, ellipsoids shown at the 20% probability level). Selected bond lengths (Å): Al(1)-Al(2) 2.660(2), Al(1)-Al(3) 2.647(2), Al(1)-Al(2)' 2.636(2), Al(1)-Al(3)' 2.632(2), Al(2)-Al(3) 2.691(2), Al(2)-Al(3)' 2.836(2), Al(1)-H(1) 1.49(5), Al(2)-H(2) 1.57(4), Al(3)-H(3) 1.59(4), Mg(1)-H(2) 1.94(4), Mg(1)-H(3)' 1.89(4). Symmetry operation: '-x+2, -y+1, -z

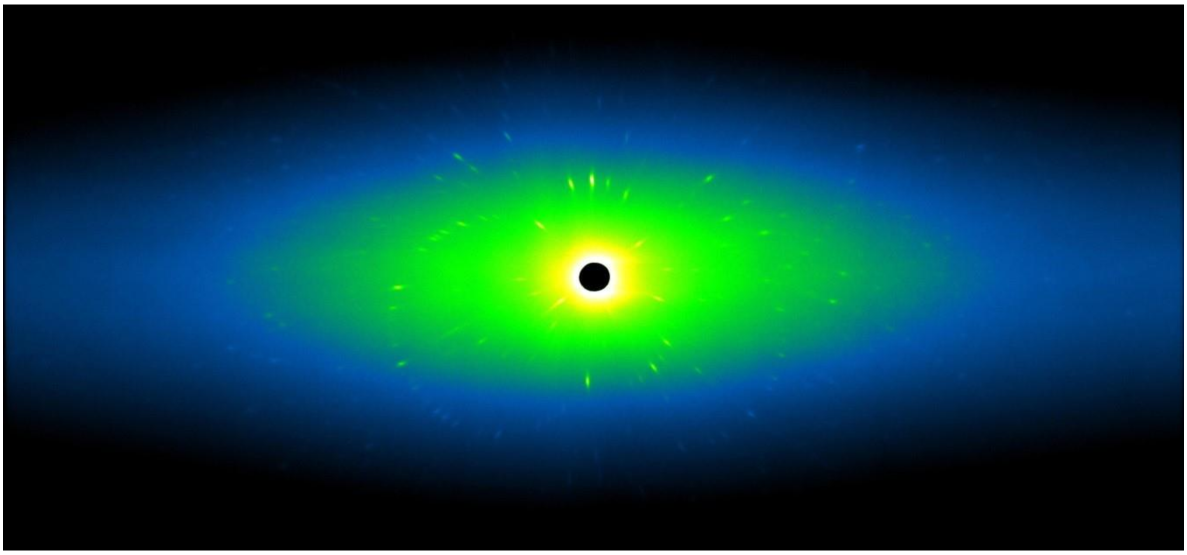

Supplementary Figure 15. A 20,000s exposure neutron diffraction image for **1a**.

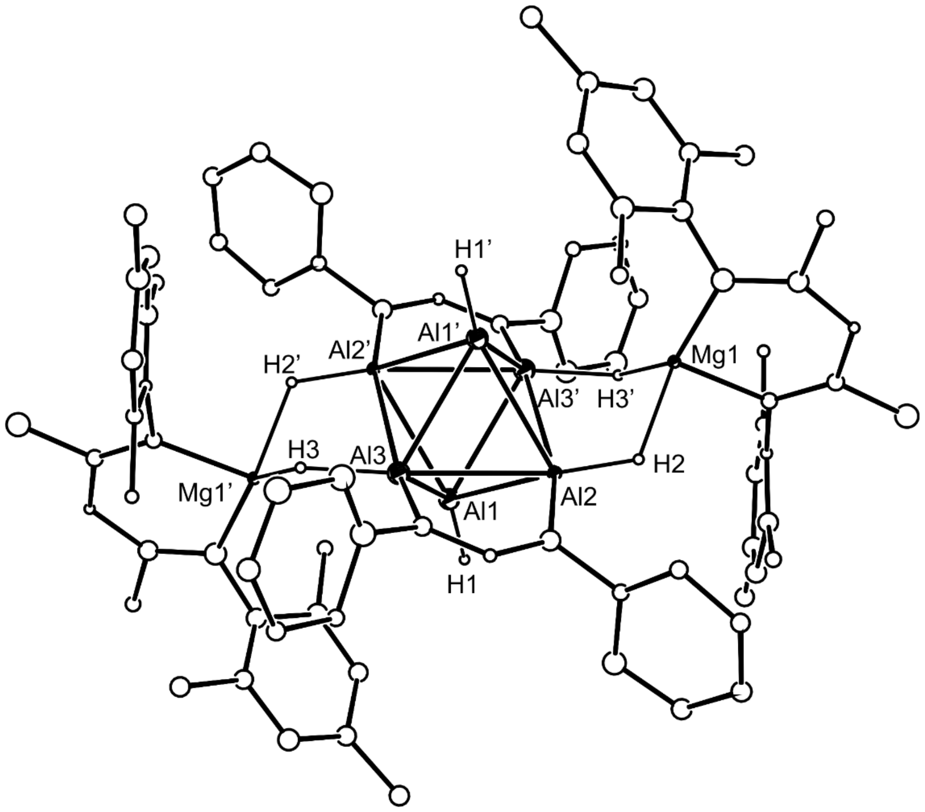

Supplementary Figure 16. Molecular structure of **1a**, as determined from the neutron diffraction study. The atom labelling scheme is that used in Fig. 2. Molecular parameters are not given due to their low precision, which arises from the low resolution of the neutron diffraction data. The portrayed molecular connectivity is, however, unambiguous.

$[(^{Me}Nacnac)Mg]_2[Al_6H_6(^{H}Fiso)_2]$

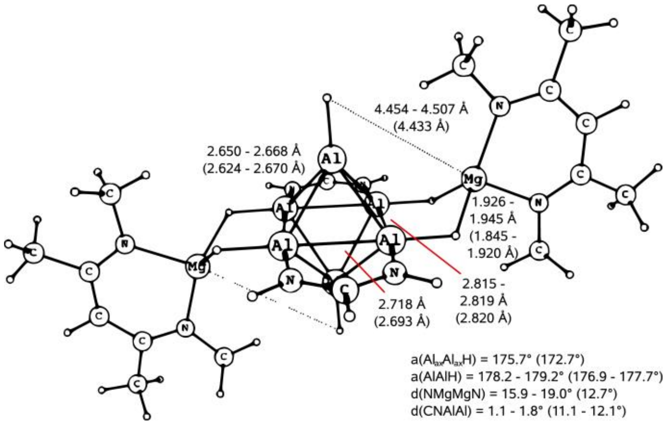

$[Al_6H_6(^{H}Fiso)_2]^{2-}$

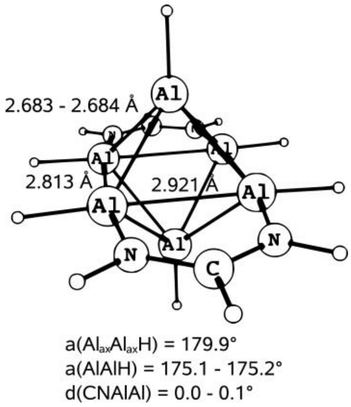

Al_6H_6 (Triplet)

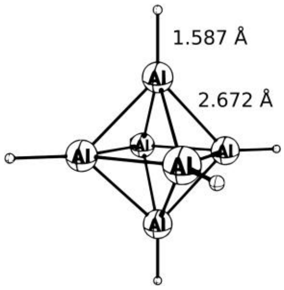

Supplementary Figure 17. Geometries of $[(^{Me}Nacnac)Mg]_2[Al_6H_6(^{H}Fiso)_2]$, $[Al_6H_6(^{H}Fiso)_2]^{2-}$ and Al_6H_6 (Triplet) optimized at RI-BP86/def2-TZVPP.

$Al_{eq4}H_{eq4}(^{H}Fiso)_2$ plane	$Al_{ax2}H_{ax2}Al_{eq2}$ plane
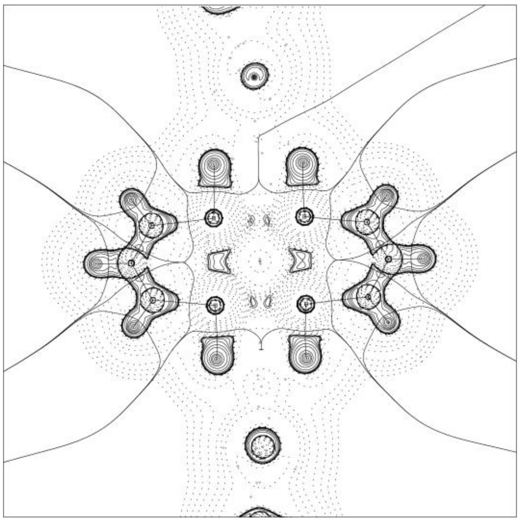	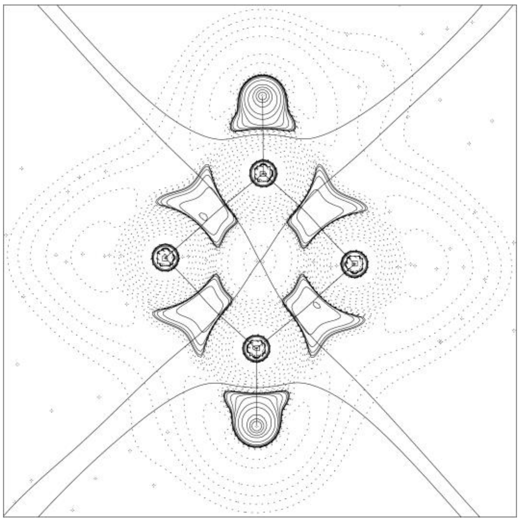

Supplementary Figure 18. Contour line diagrams $\nabla^2\rho(r)$ of $[(^{Me}Nacnac)Mg]_2[Al_6H_6(^{H}Fiso)_2]$ cut through the $Al_{eq4}H_{eq4}(^{H}Fiso)_2$ plane (left) and the $Al_{ax2}H_{ax2}Al_{eq2}$ plane (right) calculated at BP86/SVP. Solid lines indicate areas of charge concentration and dashed lines show areas of charge depletion. Thick solid lines connecting the atomic nuclei represent bond paths, thick solid lines separating the atomic basins represent the zero-flux surfaces crossing the molecular plane.

$[(^{Me}Nacnac)Mg]_2[Al_6H_6(^{H}Fiso)_2]$

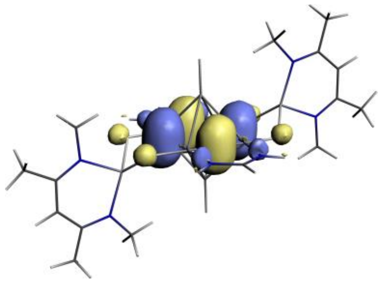

LUMO -1.53 eV

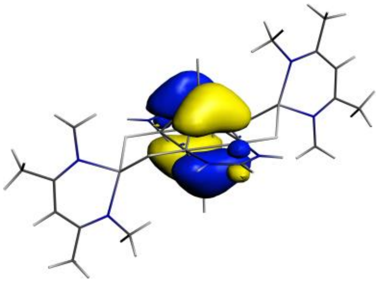

HOMO -3.69 eV

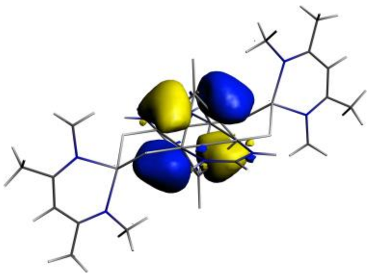

HOMO-1 -3.83 eV

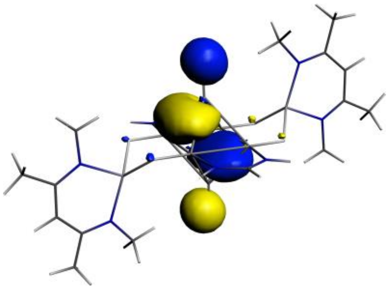

HOMO-4 -5.11 eV

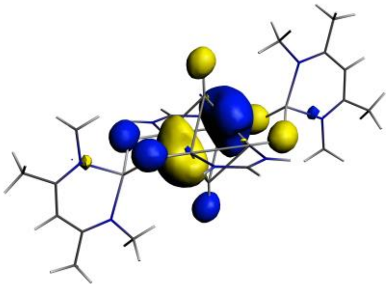

HOMO-5 -5.26 eV

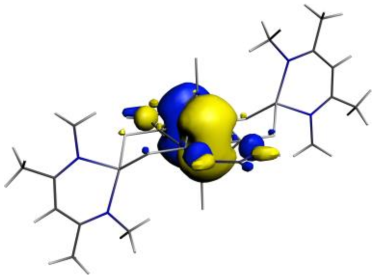

HOMO-6 -5.53 eV

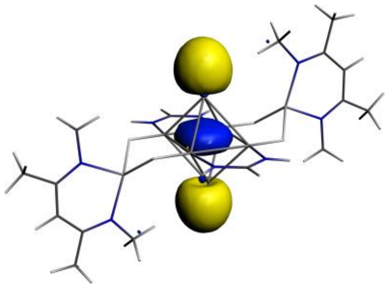

HOMO-13 -6.92 eV

Supplementary Figure 19. Molecular orbitals of $[(^{Me}Nacnac)Mg]_2[Al_6H_6(^{H}Fiso)_2]$ calculated at RI-BP86/def2-TZVPP.

$[Al_6H_6(^{H}Fiso)_2]^{2-}$

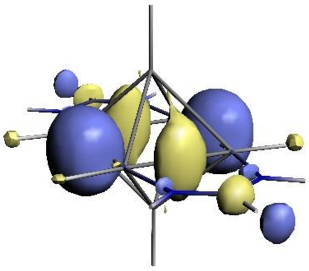

LUMO+7 5.64 eV

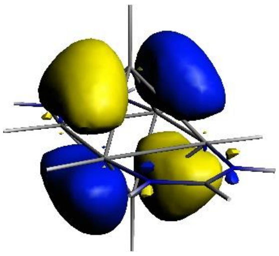

HOMO 3.42 eV

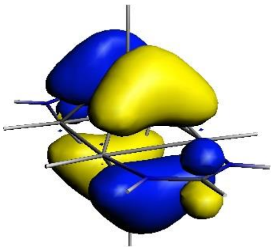

HOMO-1 3.30 eV

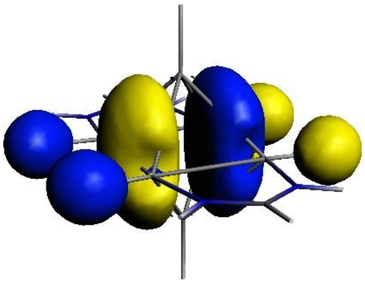

HOMO-2 1.96 eV

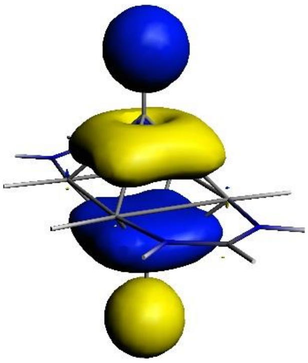

HOMO-3 1.84 eV

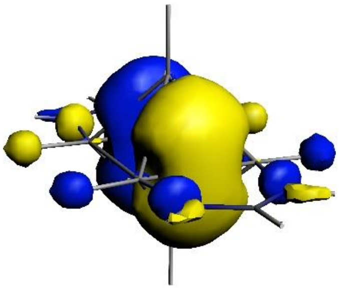

HOMO-4 1.70 eV

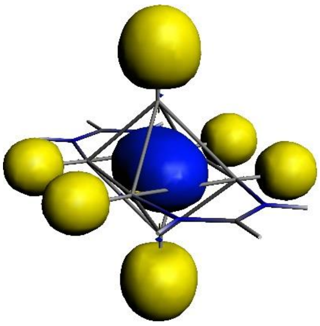

HOMO-9 -0.01 eV

Supplementary Figure 20. Molecular orbitals of $[Al_6H_6(^{H}Fiso)_2]^{2-}$ calculated at RI-BP86/def2-TZVPP.

Al$_6$H$_6$ (Triplet)

t_{2g}

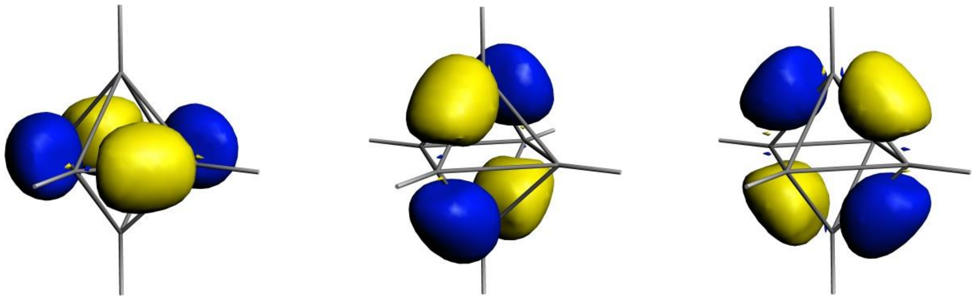

HOMO-2/SOMO1/SOMO2
α = -5.52 eV
β = -4.95 eV

t_{1u}

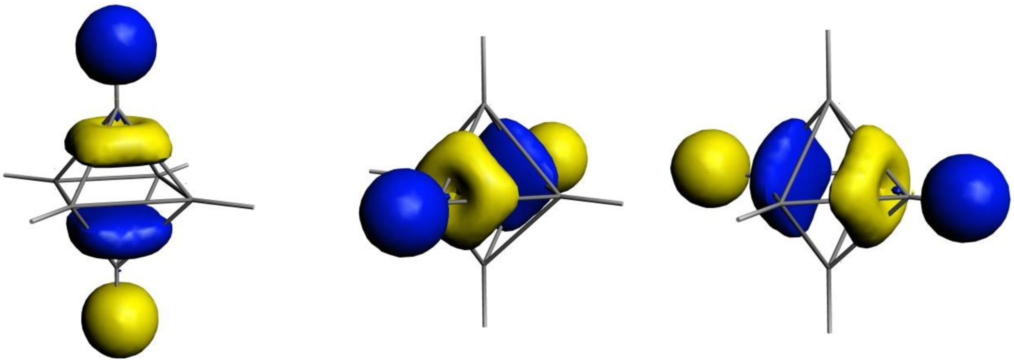

HOMO-3/4/5
α = -7.11 eV
β = -6.90 eV

a_{1g}

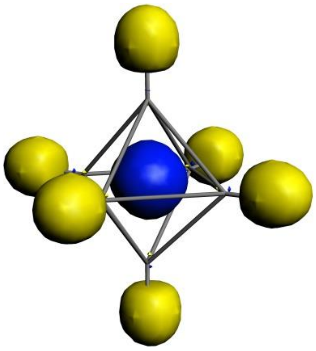

HOMO-6
α = -8.37 eV
β = -8.36 eV

Supplementary Figure 21. Molecular orbitals of Al$_6$H$_6$ (Triplet) calculated at RI-BP86/def2-TZVPP.

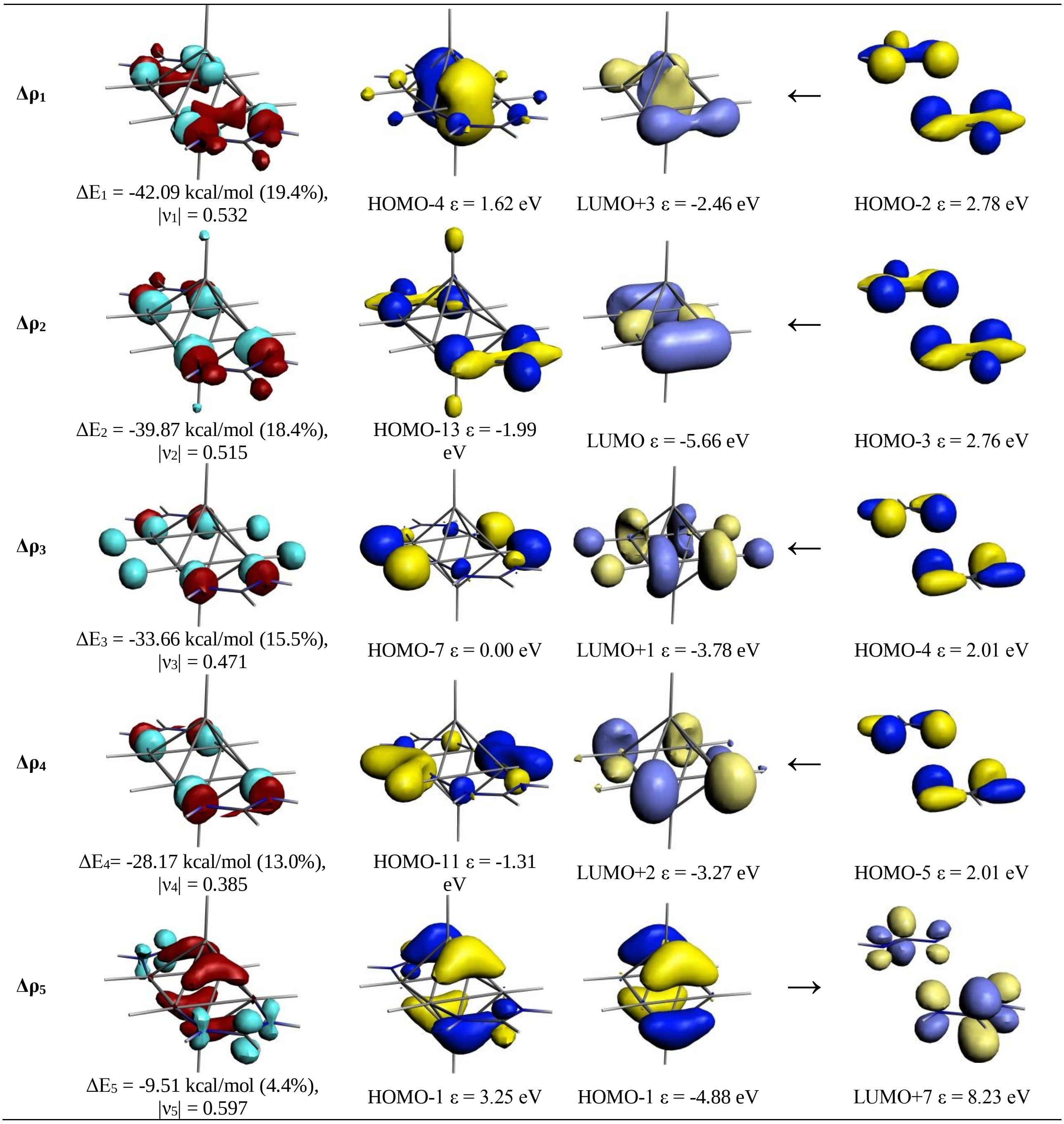

Supplementary Figure 22. Calculated (BP86/def2-TZ2P) deformation densities Δρ (left entry) of the relevant pairwise orbital interactions between Al_6H_6 and $(^{H}Fiso)_2^{2-}$ unit in $[Al_6H_6(^{H}Fiso)_2]^{2-}$. The two entries at the right hand side show the occupied and vacant orbitals of the donor and acceptor moieties. The second entry from the left shows the resulting occupied MO of the complex. The colour code for the charge flow is red→blue.

Supplementary Tables

Supplementary Table 1. Crystallographic data for **1a**·(benzene), **1b**·(hexane), **1c**·(hexane), [(^{Xyl}Nacnac)MgI(OEt$_2$)] **1S**, [{(^{Mes}Nacnac)Mg}$_2$(μ-H)]$_2$[H$_3$Al−AlH$_3$]·(benzene) **2S**·(benzene), [(Fiso)Mg(^{Dep}Nacnac)] **3S**

	**1a**·(benzene)	**1b**·(hexane)	**1c**·(benzene)	**1S**	**2S**·(benzene)	**3S**
empirical formula	$C_{102}H_{140}Al_6Mg_2N_8$	$C_{106}H_{156}Al_6Mg_2N_8$	$C_{98}H_{140}Al_6Mg_2N_8$	$C_{25}H_{35}IMgN_2O$	$C_{98}H_{130}Al_2Mg_4N_8$	$C_{50}H_{68}MgN_4$
formula weight	1688.72	1752.89	1640.68	530.76	1571.30	749.39
crystal system	triclinic	triclinic	triclinic	monoclinic	triclinic	tetragonal
space group	*P*-1	*P*-1	*P*-1	$P2_1/n$	*P*-1	$P4_32_12$
a (Å)	14.405(3)	12.6621(4)	13.8192(7)	7.8042(16)	12.3406(9)	13.2908(2)
b (Å)	14.545(3)	14.6559(5)	14.4622(7)	16.365(3)	15.5838(10)	13.2908(2)
c (Å)	15.016(3)	16.0991(6)	15.7768(9)	20.374(4)	25.0072(17)	26.0877(7)
α (º)	95.89(3)	67.185(2)	63.119(2)	90	90.457(3)	90
β (º)	115.18(3)	85.980(2)	64.272(2)	96.69(3)	98.367(3)	90
γ (º)	112.18(3)	89.488(2)	63.048(2)	90	95.075(3)	90
V (Å^3)	2483.6(9)	2746.52(16)	2400.5(2)	2584.4(9)	4738.3(6)	4608.27(16)
Z	1	1	1	4	2	4
T (K)	123(2)	123(2)	123(2)	123(2)	123(2)	185(2)
ρ_{calcd} (g×cm^3)	1.129	1.060	1.135	1.364	1.101	1.080
μ (mm^{-1})	0.126	0.116	0.128	1.281	0.105	0.075
F(000)	910	950	886	1088	1696	1632
reflns collected	16423	84107	38800	13843	22107	81950
unique reflns	8718	11913	5121	7514	16235	4046
R_{int}	0.0691	0.0605	0.0840	0.0250	0.0343	0.0443
R1 [I > 2σ(I)]	0.0618	0.0700	0.0609	0.0316	0.0584	0.0498
wR2 (all data)	0.1452	0.2108	0.1533	0.0782	0.1494	0.1172
largest peak and hole (e×Å^{-3})	0.52, -0.24	1.59, -0.48	0.62, -0.29	1.24, -0.63	0.28, -0.28	0.42, -0.35
CCDC no.	1830326	1830330	1830327	1830325	1830331	1830328

Supplementary Table 2. Coordinates for [(^{Me}Nacnac)Mg]$_2$[Al$_6$H$_6$(^{H}Fiso)$_2$], [Al$_6$H$_6$(^{H}Fiso)$_2$]$^{2-}$ and Al$_6$H$_6$ calculated at RI-BP86/def2-TZVPP.

[(^{Me}Nacnac)Mg]$_2$[Al$_6$H$_6$(^{H}Fiso)$_2$]			
Al	-1.630303	0.071121	-1.240535
Al	-1.366120	0.114503	1.463807
Al	1.422467	-0.163146	1.195299
Al	1.159300	-0.229798	-1.509212
Al	0.073699	1.736165	-0.091186
Al	-0.284187	-1.842574	0.042460
N	-3.510200	0.289429	-0.861235
H	-4.198445	0.350560	-1.608006
N	3.300427	-0.386494	0.817836
C	3.832379	-0.454960	-0.397432
N	3.081614	-0.393317	-1.491179
H	3.615024	-0.455803	-2.355001
N	-3.287316	0.293074	1.447064
C	-4.039987	0.359297	0.355180
H	3.986498	-0.446129	1.566271
H	-3.818315	0.360287	2.311624
Mg	-0.348293	0.977600	-3.828678
N	-0.700029	0.188562	-5.677042
C	-0.630075	0.918134	-6.793811
C	-0.884302	0.258960	-8.134283
C	-0.328667	2.298624	-6.820843
C	-0.064448	3.206199	-5.769757
C	0.215636	4.636075	-6.187879
N	-0.048742	2.889986	-4.468882
C	0.227989	3.957557	-3.505091
Mg	0.177464	-1.147447	3.748962
N	0.394556	-3.100719	4.293621
C	0.590830	-4.132237	3.271645
N	0.150072	-0.408669	5.653450
C	0.138757	-1.197791	6.729014
C	0.032829	-0.581400	8.109312
C	0.089323	1.039035	5.845309
C	0.367220	-3.483320	5.578051
C	0.503506	-4.954714	5.915529
C	0.226399	-2.608355	6.678229
H	-0.280166	-5.551740	5.425632
H	1.466276	-5.353976	5.562344
H	0.439497	-5.118902	6.995936
H	-0.451905	-3.437502	-0.019072
H	-1.898054	-0.167637	-8.178820
H	0.204820	-3.100793	7.648243
H	-0.186099	-0.573541	-8.307495

H	-5.125247	0.474677	0.458654
H	-0.776980	0.974969	-8.955422
H	-1.770899	0.052734	-2.901197
H	0.024278	-1.350981	8.887772
H	-1.174640	0.097508	3.113585
H	0.978737	-0.252004	-3.166707
H	-0.884286	0.018559	8.205171
H	1.562054	-0.139282	2.859398
H	-0.302433	2.737746	-7.816243
H	4.918151	-0.564317	-0.499495
H	0.873248	0.100296	8.305123
H	0.171431	4.745217	-7.275610
H	0.249283	3.329062	-0.030952
H	-0.509501	5.331349	-5.739601
H	1.209944	4.960331	-5.846972
H	-0.830787	1.363363	6.360524
H	0.944542	1.425287	6.424974
H	0.105679	1.535205	4.866580
H	0.590803	-3.666413	2.276975
H	1.550523	-4.664170	3.391066
H	-0.212197	-4.889183	3.270908
H	0.197263	3.551088	-2.485829
H	1.224387	4.408664	-3.647965
H	-0.513000	4.773036	-3.554077
C	-1.022803	-1.233425	-5.783448
H	-1.051754	-1.673937	-4.777591
H	-2.007572	-1.411870	-6.249633
H	-0.274105	-1.797465	-6.366344

$[Al_6H_6(^{H}Fiso)_2]^{2-}$

Al	-1.587603	0.039392	-1.381905
Al	-1.353684	0.006740	1.529768
Al	1.441638	-0.212450	1.303118
Al	1.207659	-0.179821	-1.608696
Al	0.064642	1.665455	-0.030200
Al	-0.210762	-1.838562	-0.048489
N	-3.500999	0.185750	-0.922096
H	-4.166120	0.245257	-1.688603
N	3.355210	-0.358495	0.843142
C	3.913594	-0.396027	-0.361710
N	3.170342	-0.331647	-1.460766
H	3.704342	-0.366943	-2.325182
N	-3.316245	0.158816	1.381836
C	-4.059359	0.223436	0.282724
H	4.020303	-0.417696	1.609672
H	-3.850340	0.194305	2.246110
H	-0.341389	-3.476930	-0.060064
H	-5.155624	0.309694	0.371747
H	-1.856186	0.065672	-3.001384
H	-1.362539	0.001989	3.170875

H	1.216447	-0.175170	-3.249899
H	1.710350	-0.238879	2.922611
H	5.009916	-0.481879	-0.450794
H	0.195477	3.303779	-0.018796
	Al_6H_6 (Triplet)		
Al	-1.6442889	0.1324634	-1.1002442
Al	-1.4479560	0.1084234	1.3503636
Al	1.6442889	-0.1324634	1.1002442
Al	1.4479560	-0.1084234	-1.3503636
Al	0.1436387	1.8362797	0.0082755
Al	-0.1436387	-1.8362797	-0.0082755
H	-0.2664210	-3.4060423	-0.0144309
H	-2.5469878	0.2077471	-2.3862474
H	-2.1342997	0.1567710	2.7649103
H	2.1342997	-0.1567710	-2.7649103
H	2.5469878	-0.2077471	2.3862474
H	0.2664210	3.4060423	0.0144309

Supplementary Table 3. IR intensities for [(^{Me}Nacnac)Mg]$_2$[Al_6H_6(^{H}Fiso)$_2$], [Al_6H_6(^{H}Fiso)$_2$]$^{2-}$ and Al_6H_6 calculated at RI-BP86/def2-TZVPP.

[(^{Me}Nacnac)Mg]$_2$[Al_6H_6(^{H}Fiso)$_2$]		
mode	wave number [cm^{-1}]	IR intensity [km/mol]
1	0.00	0.00000
2	0.00	0.00000
3	0.00	0.00000
4	0.00	0.00000
5	0.00	0.00000
6	0.00	0.00000
7	8.68	0.01803
8	10.42	0.56368
9	17.17	0.04143
10	18.83	0.97029
11	29.42	0.05060
12	39.73	0.24690
13	46.90	4.31090
14	47.89	3.20448
15	55.34	0.66968
16	60.99	0.03063
17	74.18	7.75113
18	84.37	0.06571
19	86.10	0.01942
20	86.22	0.01890
21	93.69	0.06157
22	109.72	2.64359
23	114.65	5.21058
24	118.83	0.21764
25	120.51	0.90526
26	125.93	0.13886
27	129.31	1.96563
28	133.91	0.27354
29	143.69	0.13637
30	157.95	0.00347
31	168.86	0.41520
32	182.30	0.42890
33	185.48	8.22624
34	188.98	3.17345
35	193.28	0.41589
36	195.20	0.01966
37	202.12	0.08402
38	204.03	16.98768
39	204.99	6.84436
40	209.40	1.64431
41	214.07	0.21661
42	216.58	0.01839
43	221.90	1.08246

44	229.20	0.02833
45	245.17	0.30229
46	255.34	2.84187
47	264.43	0.04527
48	268.12	0.05928
49	273.76	0.11109
50	277.94	59.30158
51	286.49	0.03578
52	297.60	46.19937
53	297.92	29.00567
54	305.10	9.35374
55	308.23	2.01726
56	312.56	0.94797
57	313.36	0.44756
58	314.70	0.48241
59	330.63	73.30569
60	341.03	0.40016
61	341.86	0.61780
62	343.07	0.07911
63	353.01	0.16057
64	355.46	5.22186
65	359.00	0.21841
66	367.08	0.01526
67	385.20	0.05004
68	388.74	6.08054
69	389.30	0.49430
70	394.11	36.17554
71	442.23	0.26552
72	450.82	233.70164
73	452.67	20.78450
74	468.23	35.41014
75	478.40	45.96047
76	484.67	19.98700
77	495.75	0.35367
78	514.68	152.18092
79	525.22	0.62772
80	539.87	1.03932
81	542.24	9.19966
82	544.79	81.89402
83	546.78	20.60469
84	549.52	5.33041
85	584.48	0.09888
86	585.29	0.17591
87	612.01	0.13434
88	612.28	0.14826
89	633.41	0.68389
90	634.80	0.54797
91	669.32	28.65673
92	669.70	5.85033
93	693.34	92.42630

94	693.64	15.85040
95	714.84	0.96894
96	727.98	16.28169
97	728.81	0.07107
98	733.52	16.02966
99	837.18	83.74787
100	838.39	15.47986
101	873.32	0.64672
102	873.78	0.51012
103	930.27	0.03047
104	931.80	0.28152
105	945.78	5.33399
106	965.20	45.13785
107	970.90	111.40109
108	974.94	23.78038
109	979.36	206.09494
110	979.69	27.86146
111	998.04	4.98363
112	999.43	4.92800
113	1006.20	2.47287
114	1010.64	2.59035
115	1012.30	0.09281
116	1013.76	0.12563
117	1052.18	28.43452
118	1052.46	35.47513
119	1069.17	1.19715
120	1071.29	0.93122
121	1077.34	0.07022
122	1080.72	0.34238
123	1097.86	9.07101
124	1098.00	0.48030
125	1117.53	1.14822
126	1120.71	1.32940
127	1123.09	4.99325
128	1125.71	5.20503
129	1135.84	23.38852
130	1136.44	1.08839
131	1219.19	46.27768
132	1219.49	1.46850
133	1251.61	12.15988
134	1253.45	14.87717
135	1269.64	47.21449
136	1271.75	38.46607
137	1338.01	133.08807
138	1339.72	1.55434
139	1349.59	15.73758
140	1351.74	18.17820
141	1353.53	15.45755
142	1354.66	18.26796
143	1379.54	172.64686

144	1382.24	150.19210
145	1400.37	81.03824
146	1403.20	65.57142
147	1424.13	4.12985
148	1425.18	9.88353
149	1425.47	38.83855
150	1426.24	5.80577
151	1427.58	3.53112
152	1428.34	53.45604
153	1428.99	4.22522
154	1429.38	2.54227
155	1434.73	99.25504
156	1438.70	84.70630
157	1439.13	12.04828
158	1440.75	11.96275
159	1448.41	10.28507
160	1450.65	15.55012
161	1453.66	2.22003
162	1453.95	11.43919
163	1454.51	2.97059
164	1456.75	7.86802
165	1485.54	96.62041
166	1485.65	118.37226
167	1524.46	457.52194
168	1524.98	174.98926
169	1530.08	864.06569
170	1532.30	99.73645
171	1576.17	119.04630
172	1583.77	334.03465
173	1591.46 (Al_{eq})-H	3.97651
174	1614.87 (Al_{eq})-H	106.53143
175	1649.11 (Al_{eq})-H	1402.81548
176	1670.04 (Al_{eq})-H	51.72030
177	1797.73 (Al_{ax})-H(asymm.)	449.76036
178	1805.78 (Al_{ax})-H(symm.)	34.62453
179	2910.51	84.15198
180	2911.84	82.93746
181	2916.88	51.30130
182	2917.93	78.93885
183	2955.03	25.75711
184	2960.44	21.48147
185	2960.46	20.14473
186	2962.13	18.45386
187	2962.60	26.47209
188	2964.33	16.80449
189	2964.65	18.78283
190	2964.66	23.77703
191	3009.40	9.18549
192	3010.60	8.69918
193	3011.77	4.91662

194	3012.66	9.52326
195	3013.97	9.93006
196	3015.38	25.09015
197	3016.86	2.98331
198	3024.39	24.93315
199	3036.99	21.12157
200	3039.17	15.98522
201	3064.70	26.96144
202	3064.74	15.77036
203	3066.79	11.13184
204	3070.85	17.47916
205	3117.65	23.08076
206	3119.37	21.68349
207	3500.17	27.37035
208	3502.57	32.77476
209	3504.15	22.52315
210	3506.38	25.42730

$[Al_6H_6(^{H}Fiso)_2]^{2-}$

mode	wave number [cm^{-1}]	IR intensity [km/mol]
1	0.00	0.00000
2	0.00	0.00000
3	0.00	0.00000
4	0.00	0.00000
5	0.00	0.00000
6	0.00	0.00000
7	70.19	26.06625
8	75.48	0.00303
9	101.48	9.56844
10	112.24	0.00457
11	137.32	3.65142
12	147.52	0.00000
13	177.06	0.00234
14	182.37	0.00000
15	183.69	0.00000
16	197.07	0.00000
17	221.18	0.00000
18	239.25	0.00000
19	243.05	1.83811
20	264.14	0.00000
21	274.85	0.65634
22	279.65	0.16215
23	290.76	5.81505
24	304.84	0.00000
25	333.79	0.00000
26	348.95	0.00000
27	353.67	42.12275

28	355.41	0.00015
29	360.84	0.00004
30	376.00	0.00000
31	432.95	35.33524
32	443.18	0.02631
33	457.94	0.00049
34	464.64	23.94532
35	476.44	0.00007
36	487.69	122.60084
37	509.34	27.66904
38	513.61	84.94442
39	530.50	0.00001
40	534.05	0.00657
41	583.84	284.29381
42	617.26	148.39992
43	627.87	0.00060
44	633.02	0.00011
45	674.53	0.02062
46	675.76	109.44321
47	741.88	43.99327
48	758.18	0.00005
49	849.16	0.00134
50	851.15	1.98596
51	1107.82	144.64146
52	1109.62	0.00039
53	1164.53	41.10998
54	1165.02	0.21551
55	1312.28	70.82708
56	1314.38	0.00072
57	1380.08	43.47325
58	1384.75	0.00098
59	1582.56	297.92050
60	1586.82	0.08502
61	1617.86	1188.23913
62	1623.01	198.55555
63	1631.16	0.01201
64	1633.26	0.19865
65	1643.35	1625.07670
66	1664.67	0.00339
67	2930.43	257.29113
68	2931.32	12.13907
69	3496.53	2.05392
70	3496.88	3.89017
71	3498.04	0.23536
72	3498.45	0.07549

Al_6H_6 (Triplet)

mode	wave number [cm^{-1}]	IR intensity [km/mol]
1	-0.00	0.00000

2	0.00	0.00000
3	0.00	0.00000
4	0.00	0.00000
5	0.00	0.00000
6	0.00	0.00000
7	76.12	0.00610
8	83.11	0.00000
9	180.35	0.00000
10	191.18	0.00118
11	213.91	1.38932
12	238.81	0.00000
13	242.10	0.63090
14	266.71	9.85972
15	276.70	0.00000
16	313.06	0.00000
17	321.61	49.33747
18	397.27	0.00000
19	401.52	70.66225
20	409.32	0.00000
21	413.31	0.00000
22	414.55	17.69696
23	430.63	0.00000
24	448.95	0.00141
25	463.95	0.00000
26	468.10	0.00000
27	483.49	0.00000
28	501.30	36.42275
29	509.55	11.88408
30	594.32	100.05063
31	1995.46	631.93742
32	1996.32	0.00000
33	1997.77	478.03409
34	1997.90	0.00000
35	2000.44	744.39704
36	2014.43	0.00000

Supplementary Table 4. NBO atomic partial charges calculated at BP86/def2-TZVPP for [(^{Me}Nacnac)Mg]$_2$ [Al_6H_6(^{H}Fiso)$_2$] and [Al_6H_6(^{H}Fiso)$_2$]$^{2-}$.

[e]	[(^{Me}Nacnac)Mg]$_2$[Al_6H_6(^{H}Fiso)$_2$]	[Al_6H_6(^{H}Fiso)$_2$]$^{2-}$
Al_6H_6	-0.668	-0.627
[(^{Me}Nacnac)Mg]$_2$	1.763	
(^{H}Fiso)$_2$	-1.095	-1.373
H_{ax}	-0.286–(-0.285)	-0.331
H_{eq}	-0.436–(-0.434)	-0.363
Al_{ax}	-0.059–(-0.054)	-0.189
Al_{eq}	0.438–0.443	0.466
Mg	1.716–1.717	

Supplementary Table 5. Wiberg bond indices calculated at BP86/def2-TZVPP for [(^{Me}Nacnac)Mg]$_2$ [Al_6H_6(^{H}Fiso)$_2$] and [Al_6H_6(^{H}Fiso)$_2$]$^{2-}$.

	[(^{Me}Nacnac)Mg]$_2$[Al_6H_6(^{H}Fiso)$_2$]	[Al_6H_6(^{H}Fiso)$_2$]$^{2-}$
Al_{ax}-Al_{eq}	0.63 – 0.66	0.66
Al_{eq}-Al_{eq} (^{H}Fiso)	0.32	0.23
Al_{eq}-Al_{eq} ((^{Me}Nacnac)Mg)	0.30	0.30
Al_{eq}-H_{eq}	0.65	0.79
Al_{ax}-H_{ax}	0.87	0.84
Mg- H_{eq}	0.01	
Mg-H_{ax}	0.00	
Al_{ax}-Mg	0.06	

Supplementary Table 6. EDA-NOCV results at the BP86/def2-TZ2P level for $[Al_6H_6(^{H}Fiso)_2]^{2-}$ with Al_6H_6 and $(^{H}Fiso)_2^{2-}$ as interacting fragments. Energy values in kcal mol^{-1}.

[kcal/mol]	Al_6H_6 + $(^{H}Fiso)_2^{2-}$
ΔE_{int}	-239.77
ΔE_{Pauli}	371.54
ΔE_{elstat}	-394.58 (64.6%)
ΔE_{orb}	-216.74 (35.5%)

Supplementary Table 7. SCF GIAO Magnetic shielding tensor and (in parentheses) NICS values at a reference point placed in the centre of the Al_6H_6 moiety of $[(^{Me}Nacnac)Mg]_2[Al_6H_6(^{H}Fiso)_2]$ calculated at BP86/def-TZVPP. Z axis is perpendicular to Al_4 plane, x axis goes through nitrogen atoms of $^{H}Fiso_2$ and y axis goes through Mg atoms.

[ppm]	SCF GIAO Magnetic shielding tensor (NICS value)
Isotropic	12.49 (-12.49)
Anisotropy	50.20
XX	-2.02
XY	0.29
XZ	-0.44
ZX	-0.40
ZY	4.60
ZZ	45.74 (-45.74)

Supplementary Methods

General synthetic procedures. All manipulations were carried out using standard Schlenk and glove box techniques under an atmosphere of high purity nitrogen. Hexane, cyclohexane, THF and toluene were distilled over potassium, whilst diethyl ether was distilled over Na/K alloy. ^{1}H and $^{13}C\{^{1}H\}$ NMR spectra were recorded on either a Bruker DPX 300 or AvanceIII 400 spectrometers and were referenced to the resonances of the solvent used. Solid state $^{13}C\{^{1}H\}$ NMR spectra were recorded on a Bruker DPX 400 spectrometer and were referenced to glycine. EI mass spectra were obtained from the EPSRC National Mass Spectrometric Service at Swansea University, UK. MALDI-TOF spectra were recorded on a Voyager-DE STR Biospectrometry Workstation. IR spectra were recorded using a Perkin Elmer 1600 FT-IR spectrometer as Nujol mulls between NaCl plates. Raman spectra were recorded for crystalline samples sealed in glass capillaries under dinitrogen using a Renishaw RM2000 micro-Raman spectrometer with 514 nm excitation from a diode laser. Melting points were determined in sealed glass capillaries under dinitrogen and are uncorrected. Microanalyses were performed by the Campbell Microanalytical Laboratory, University of Otago, Dunedin, New Zealand, or at the Science Centre, London Metropolitan University. The starting material FisoH[1] was prepared by literature procedures, whilst all other reagents were purchased from the Aldrich Chemical Company and used as received.

Preparation of FisoD.DCl: FisoH (2.00 g, 5.46 mmol) was dissolved in dry toluene (50 mL). D_2O (0.20 g, 12.02 mmol) and $SiMe_3Cl$ (1.63 g,15.0 mmol) were added to the solution at -78 °C, the reaction vessel sealed, then warmed to room temperature. The resultant solution was stirred at room temperature overnight before the volatiles were removed *in vacuo*. Toluene (30 mL) was added to the residue and the ensuing slurry was dried with $SiMe_3Cl$ (1.63 g, 15.00 mmol). Volatiles were again removed under reduced pressure and the residue was used without further purification (yield 90%). ^{1}H NMR (400 MHz, 298 K, $CDCl_3$) = 1.20 (d, $^{3}J_{H,H}$ = 6.8 Hz, 24H; $CH(CH_3)_2$), 3.20 (sept, $^{3}J_{H,H}$ = 6.8 Hz, 4H; $CH(CH_3)_2$), 7.09-7.43 (m, 7H; NC*H*N and Ar-*H*). For synthetic details of FisoH.HCl see ref. 2.

Preparation of [{(μ-*N*,*N*-Fiso)Al(D)(μ-D)}$_2$]: The compound was synthesised according to the procedure reported for [{(μ-*N*,*N*-Fiso)Al(H)(μ-H)}$_2$][2]. Diethyl ether (30 mL) was added to FisoD.DCl (0.75 g, 1.86 mmol) and the resulting slurry cooled to −78 °C. A precooled

(−78 °C) diethyl ether (20 mL) solution of $LiAlD_4$ (100 mg, 2.38 mmol) was slowly added to the slurry. The mixture was then slowly warmed to room temperature, and stirred for 2 d. Volatiles were removed *in vacuo* and the residue extracted with toluene (40 mL). The extract was concentrated under reduced pressure to incipient crystallisation. Storing the solution at −30 °C overnight resulted in the deposition of colourless crystals of the title compound (yield = 0.19 g, 10%). The spectroscopic data for the compound are identical to those for [{(μ-*N*,*N*-Fiso)Al(H)(μ-H)}$_2$] with the exception of a missing hydride resonance in the ^{1}H NMR spectrum of the compound, and missing Al-H stretching modes in its infrared spectrum[2].

X-ray crystallographic studies. Crystals of **1a**·(benzene), **1b**·(hexane), **1c**·(hexane), [(^{Xyl}Nacnac)MgI(OEt_2)] **1S**, [{(^{Mes}Nacnac)Mg}$_2$(μ-H)]$_2$[H_3Al–AlH_3]·(benzene) **2S**·(benzene), [(Fiso)Mg(^{Dep}Nacnac)] **3S** suitable for X-ray structural determinations were mounted in silicone oil. Crystallographic measurements were made using a Bruker Apex X8 diffractometer using MoK_α radiation (λ = 0.71073 Å). The structures were solved by direct methods and refined on F^2 by full matrix least squares (SHELX97)[3] using all unique data. Hydrogen atoms are typically included in calculated positions (riding model), except for the hydride ligands of **1a-c**, and **3S**, the positional and atomic displacement parameters of which were freely refined, isotropically. The Flack parameter for the structure of **3S** refined to 0.0(4). The low 2θ angle limit of data used for the refinement of the structure of **1c** is due to diffraction data being extremely weak above this angle. Despite this, the complex is isostructural to **1a** and **1b**, and hence the molecular connectivity of the compound is unambiguous. The largest residual electron density peak (1.59 e-/Å^3) in the structure of **1b** arises due to minor disorder in the hexane of solvation, which could not be successfully modelled.

Neutron diffraction study. A crystal of **1a** of approximate dimensions 2 x 2 x 1mm was mounted on the Koala single crystal neutron diffraction instrument at ANSTO, Lucas Heights, Sydney. The quality and size of the sample dictated that the collected diffraction data were of relatively low resolution (see Supplementary Figure 15). Indexing of the diffraction pattern from the first long exposure of the crystal was readily achieved using the LAUEG software[4], but the extraction of useful data from the patterns required manual intervention in the integration[5] and normalization[4] processes embedded in the software.

The data-set obtained could be employed to determine the presence, locus and occupancy of all of the hydrogen atoms in the structure. Of 2297 reflections integrated for the wavelength range 0.85 – 1.70Å to a *d*-spacing of 1.0Å, 1078 independent data $I \geq 3\ \sigma I$ were used in the modelling of the structure. With excellent X-ray diffraction studies in hand, the need for neutron diffraction lay in the independent derivation of the location of the hydrides. Modelling commenced from the X-ray derived non-hydrogen atom model of the same formulation and an initial round of Fourier refinement demonstrated suitable fit of the starting model to the data. Calculation of a difference map revealed all H-atom sites of the structure, with the three crystallographically unique hydrides (one half of a molecule per asymmetric unit) being observed within the ten highest magnitude difference peaks along with other geometrically constrained hydrogen atoms (aryl and similar). Fully occupied but less constrained H-atoms were observed at lesser magnitudes with half occupied disordered methyl H atoms clearly evidenced in the map by the lowest magnitude difference peaks but clearly differentiated from the background.

A full-matrix least-squares refinement of the structure against the available data was achieved by employing positional and isotropic displacement parameters for all atoms together with application of an appropriate set of similarity and planarity restraints to the geometry of the organic ligands (Supplementary Figure 16). Although clearly evident in the difference map, no modelling of the methyl group disorder was included in the final model.

The unambiguous determination of the loci of the hydride ligands from the "negative peaks" is definitive even from such low resolution data. The imprecision of the hydride bonding geometry arises directly from the acknowledged limitations of the data set (and associated uncertainties) available from the only crystal of the compound for which neutron diffraction was achieved.

The definition of the H-atom sites from this low resolution study confirms the observations of hydride sites in the X-ray diffraction experiments and provides important observations against which the computations could be validated.

Theoretical Methods.

Geometry optimizations have been carried out using TurboMole 6.1 optimizer[6] and gradients at the RI-BP86/def2-TZVPP level of theory[7-10]. Stationary points were characterized as minima by calculating the Hessian matrix analytically at this level of theory. The QTAIM[11] calculations were performed with the AIMPAC program[12] at the

BP86/SVP//RI-BP86/def2-TZVPP level. The NICS[13] and NBO[14] calculations version 6.0 were carried out at BP86/TZVPP//RI-BP86/TZVPP with the program Gaussian 16[15].

The energy decomposition analysis with the EDA method was carried out with the program package ADF13.01c[16-17]. BP86 was chosen applying uncontracted Slater-type orbitals (STOs) as basis functions[18]. The latter basis sets for all elements have triple-ζ quality augmented by two sets of polarization functions (ADF-basis set TZ2P). This level of theory is denoted BP86/TZ2P.

The energy decomposition analysis (EDA) was developed by Ziegler and Rauk[19]. The bonding analysis focuses on the instantaneous interaction energy ΔE_{int} of a bond A–B between two fragments A and B in the particular electronic reference state and in the frozen geometry of AB. This interaction energy is divided into three main components [Eq. (1)].

$$\Delta E_{int} = \Delta E_{elstat} + \Delta E_{Pauli} + \Delta E_{orb} \qquad (1)$$

The term ΔE_{elstat} corresponds to the quasi-classical electrostatic interaction between the unperturbed charge distributions of the prepared atoms and is usually attractive. The Pauli repulsion ΔE_{Pauli} is the energy change associated with the transformation from the superposition of the unperturbed electron densities $\rho_A + \rho_B$ of the isolated fragments to the wavefunction $\Psi^0 = N\hat{A}[\Psi_A\Psi_B]$, which properly obeys the Pauli principle through explicit antisymmetrization ($\hat{A}$ operator) and renormalization (N = constant) of the product wavefunction. ΔE_{Pauli} comprises the destabilising interactions between electrons of the same spin on either fragment. The orbital interaction ΔE_{orb} accounts for charge transfer and polarization effects. The ΔE_{orb} term can be decomposed into contributions from each irreducible representation of the point group of the interacting system.

The EDA-NOCV method[20,21] combines charge (NOCV) and energy (EDA) decomposition schemes to decompose the deformation density which is associated with the bond formation, Δρ, into different components of the chemical bond. The EDA-NOCV calculations provide pair wise energy contributions for each pair of interacting orbitals to the total bond energy. NOCV (Natural Orbital for Chemical Valence)[22,23] is defined as the eigenvector of the valence operator, $\hat{V}$, given by Equation (2):

$$\hat{V}\Psi_i = \upsilon_i \Psi_i \qquad (2)$$

In the EDA-NOCV scheme the orbital interaction term, ΔE_{orb}, is given by Equation (3):

$$\Delta E_{orb} = \sum_k \Delta E_k^{orb} = \sum_{k=1}^{N/2} \upsilon_k \left[-F_{-k,-k}^{TS} + F_{k,k}^{TS}\right] \qquad (3)$$

In which $F_{-k,-k}^{TS}$ and $F_{k,k}^{TS}$ are diagonal transition state Kohn-Sham matrix elements corresponding to NOCVs with the eigenvalues $-\nu_k$ and ν_k, respectively. The ΔE_k^{orb} term of a particular type of bond are assigned by visual inspection of the shape of the deformation density, $\Delta\rho_k$. For further details of the EDA-NOCV method see recent review articles[24-28].

Supplementary References

1. Cole, M. L. & Junk, P. C. *J. Organomet. Chem.* **666**, 55– 62 (2003).
2. Cole, M. L., Jones, C., Junk, P. C., Kloth, M., Stasch, A. *Chem. Eur. J.* **11**, 4482-4491 (2005).
3. Sheldrick, G. M. *SHELX-97*, University of Göttingen, 1997.
4. Piltz, R. *Acta Cryst.* **A67**, C155 (2011).
5. Wilkinson, C., Khamis, H. W., Stansfield, R. F. D. & McIntyre, G. J. *J. Appl. Cryst.* **21**, 471-478 (1988).
6. Ahlrichs, R., Bär, M., Häser, M., Horn, H. & Kölmel, C. *Chem. Phys. Lett.* **162**, 165-169 (1989).
7. Eichhorn, K., Treutler, O., Ohm, H., Häser, M. & Ahlrichs, R. *Chem. Phys. Lett.* **242**, 652-660 (1995).
8. Becke, A. D. *Phys. Rev. A* **38**, 3098-3100 (1988).
9. Perdew, J. P. *Phys. Rev. B* **33**, 8822-8824 (1986).
10. Weigend, F. & Ahlrichs, R. *Phys. Chem. Chem. Phys.* **7**, 3297-3305 (2005).
11. Bader, R. F. W. *Atoms in Molecules: A Quantum Theory* (Oxford University Press, Oxford, 1990).
12. The program was developed by the group of Prof. Bader and was downloaded from: http://www.chemistry.mcmaster.ca/aimpac/.
13. Schleyer, P. v. R., Maerker, C., Dransfeld, A., Jiao, H. & von Hommes, N. J. R. *J. Am. Chem. Soc.* **118**, 6317-6318 (1996).
14. Reed, A. E., Weinstock, R. B. & Weinhold, F. *J. Chem. Phys.* **83**, 735-746 (1985).
15. Gaussian 16, Revision B.01, Frisch, M. J. *et al.* (Gaussian, Inc., Wallingford CT, 2016).
16. Te Velde, G. *et al. J. Comput. Chem.* **22**, 931-967 (2001).
17. *Computer code ADF 2013.01: see http://www.scm.com*.
18. Van Lenthe, E. & Baerends, E. J. *J. Comput. Chem*. **24**, 1142-1156 (2003).
19. Ziegler, T. & Rauk, A. *Inorg. Chem.* **18**, 1755-1759 (1979).
20. Mitoraj, M. P., Michalak, A. & Ziegler, T. *J. Chem. Theory Comput.* **5**, 962-975 (2009).
21. Mitoraj, M. P., Michalak, A. & Ziegler, T. *J. Phys. Chem. A* **112**, 1933-1939 (2008).
22. Mitoraj, M. P. & Michalak, A. *Organometallics* **26**, 6576-6580 (2007).

23. Mitoraj, M. P. & Michalak, A. *J. Mol. Model.* **14**, 681-687 (2008).

24. Zhao, L., von Hopffgarten, M., Andrada, D. M. & Frenking, G. *WIREs Comput. Mol. Sci.*, advance article online DOI:10.1002/wcms.1345.

25. Zhao, L., Hermann, M., Holzmann, N. & Frenking, G. *Coord. Chem. Rev.* **344**, 163-204 (2017).

26. Frenking, G. *et al. Chem. Soc. Rev.* **43**, 5106-5139 (2014).

27. Frenking, G., Hermann, M., Andrada, D. M. & Holzmann, N. *Chem. Soc. Rev.* **45**, 1129-1144 (2016).

28. Frenking, G. & Bickelhaupt F. M. in, *The Chemical Bond 1. Fundamental Aspects of Chemical Bonding*, Frenking, G.; Shaik, S., Eds. (Wiley-VCH, Weinheim, 2014) pp 121-158.
